# Immune-Related Gene-Based Novel Subtypes to Establish a Model Predicting the Risk of Prostate Cancer

**DOI:** 10.3389/fgene.2020.595657

**Published:** 2020-11-13

**Authors:** Enchong Zhang, Jieqian He, Hui Zhang, Liping Shan, Hongliang Wu, Mo Zhang, Yongsheng Song

**Affiliations:** ^1^Department of Urology, Shengjing Hospital of China Medical University, Shenyang, China; ^2^Department of Spine and Joint Surgery, Shengjing Hospital of China Medical University, Shenyang, China

**Keywords:** prostate cancer, immune, subtype, machine learning, prognosis, multi-omics

## Abstract

**Background:**

There is significant heterogeneity in prostate cancer (PCa), but immune status can reflect its prognosis. This study aimed to explore immune-related gene-based novel subtypes and to use them to create a model predicting the risk of PCa.

**Methods:**

We downloaded the data of 487 PCa patients from The Cancer Genome Atlas (TCGA) database. We used immunologically relevant genes as input for consensus clustering and applied survival analysis and principal component analysis to determine the properties of the subtypes. We also explored differences of somatic variations, copy number variations, *TMPRSS2-ERG* fusion, and androgen receptor (AR) scores among the subtypes. Then, we examined the infiltration of different immune cells into the tumor microenvironment in each subtype. We next performed Gene Set Enrichment Analysis (GSEA) to illustrate the characteristics of the subtypes. Finally, based on the subtypes, we constructed a risk predictive model and verified it in TCGA, Gene Expression Omnibus (GEO), cBioPortal, and International Cancer Genome Consortium (ICGC) databases.

**Results:**

Four PCa subtypes (C1, C2, C3, and C4) were identified on immune status. Patients with the C3 subtype had the worst prognosis, while the other three groups did not differ significantly from each other in terms of their prognosis. Principal component analysis clearly distinguished high-risk (C3) and low-risk (C1 + 2 + 4) patients. Compared with the case in the low-risk subtype, the Speckle-type POZ Protein (*SPOP*) had a higher mutation frequency and lower transcriptional level in the high-risk subtype. In C3, there was also a higher frequency of copy number alterations (CNA) of Clusterin (*CLU*) and lower *CLU* expression. In addition, C3 had a higher frequency of *TMPRSS2-ERG* fusion and higher AR scores. M2 macrophages also showed significantly higher infiltration in the high-risk subtype, while CD8^+^ T cells and dendritic cells had significantly higher infiltration in the low-risk subtype. GSEA revealed that MYC, androgen, and KRAS were relatively activated and p53 was relatively suppressed in high-risk subtype, compared with the levels in the low-risk subtype. Finally, we trained a six-gene signature risk predictive model, which performed well in TCGA, GEO, cBioPortal, and ICGC databases.

**Conclusion:**

PCa can be divided into four subtypes based on immune-related genes, among which the C3 subtype is associated with a poor prognosis. Based on these subtypes, a risk predictive model was developed, which could indicate patient prognosis.

## Introduction

Prostate cancer (PCa) is the most common cancer found in men and ranks second among the causes of cancer-related deaths in males in the United States (Crea, 2019; Siegel et al., 2019). According to the latest global cancer data^1^ of the World Health Organization (WHO), among men, the age-standardized rate (ASR) of PCa ranks second, its 5-year prevalence is the highest globally, and its age-standardized mortality is the sixth highest. PCa has a high degree of heterogeneity, which leads to different prognoses in patients after treatment (Chang et al., 2014; Peng et al., 2018). Clinicians mainly employ the pathological preoperative prostate-specific antigen (PSA), Gleason score, and clinical staging to estimate the prognosis of PCa patients (Eminaga et al., 2018; Pernar et al., 2018). However, these clinicopathological variables do not have satisfactory specificity and sensitivity in estimating the prognosis of such patients (Peng et al., 2018). Therefore, there is a need to explore the subtypes of PCa and develop an effective risk predictive model.

The immune system is the guardian of the body’s health, protecting us from infectious diseases, other foreign invaders, and internal dysfunctions, such as microbes and cancers. Cancer immunoediting refers to the three stages by which tumors evade the immune system, namely, elimination, equilibrium, and escape (Pardoll, 2012). Changing the interaction of various immune cells in the tumor microenvironment can promote this process (Hanahan and Weinberg, 2011; Heinrich et al., 2012). All cancers undergo immunoediting and are clinically detected during the escape phase. Natural killer cells, macrophages, polymorphonuclear cells, T cells, dendritic cells, and B cells constitute the tumor microenvironment. High mutation rates and genetic instability lead to increased production of new epitopes, which induce a multiphenotypic immune response and produce a tumor microenvironment of chronic inflammation (Alexandrov et al., 2013; Shalapour and Karin, 2015). Increasing evidence has shown the anticancer effect of the host immune system, which has promoted the application of different immunotherapeutic drugs in clinical trials, leading to significant progress in the diagnosis and treatment of cancer (Zheng et al., 2018; Lingohr et al., 2019). PCa is a known immunogenic disease, which can escape the immune system by downregulating human leukocyte antigen class I and thereby rendering antigen presentation ineffective. This is achieved by inducing T-cell apoptosis through the expression of the Fas ligand, by secreting immunosuppressive cytokines such as TGF-β or by increasing regulatory T cells (Tregs) (Drake et al., 2006; Drake, 2010). Several studies have shown that the combination of checkpoint inhibitors or cancer vaccines with different immunotherapeutic agents, radiation therapy (radium 223), hormonal therapy (enzalutamide), chemotherapy (docetaxel), or DNA-damaging agents (olaparib) can enhance immune responses and induce more dramatic, long-lasting clinical responses without obvious toxicity (Bilusic et al., 2017). Therefore, we may be able to further explore the biological mechanism of PCa and better help to predict the prognosis of patients by reclassifying the subtypes of tumors through differences in immune status.

Machine learning methods can automatically learn from a large scale of training data and capture signals to make accurate decisions. There have been many significant studies using machine learning to predict the prognosis of PCa patients. One 2019 study introduced a method that uses machine learning techniques to identify transcripts that correlate with PCa development and progression (Alkhateeb et al., 2019). Another interesting study used a novel machine learning method to analyze gene expression of PCa with different Gleason scores and identify potential genetic biomarkers for each Gleason group (Hamzeh et al., 2019). In this study, we obtained genes related to immune status from the IMMPORT database^2^ (Bhattacharya et al., 2018). Consensus clustering is a popular method of searching tumor genomes and is often used to discover new molecular subtypes of tumors (Monti et al., 2003). In this study, we used the expression of immunologically relevant genes as input for the consensus clustering to obtain novel molecular subtypes of PCa and to construct a prognostic risk prediction model for patients based on this subtype classification.

## Materials and Methods

### Data Acquisition

The Cancer Genome Atlas (TCGA), a landmark cancer genomics program, molecularly characterized over 20,000 primary cancer and matched normal samples spanning 33 cancer types (Blum et al., 2018). We downloaded the RNA-seq data of 497 PCa tissues and 52 normal prostate tissues in TCGA database (Blum et al., 2018). The RNA-seq data are in the form of HTSeq-Counts and HTSeq-FPKM. We converted the RNA-seq data in FPKM into RNA-seq data in TPM. A total of 60,483 genes were included in the RNA-seq data. We extracted 19,463 protein-coding genes from these 60,483 genes using gene annotations from the Ensembl database^3^ (Yates et al., 2020). We also downloaded the simple nucleotide variation data in TCGA, along with the copy number variation data of PCa. Then, we downloaded the XML files containing the clinical information of 498 patients. We sorted through the clinical information of the patients and eliminated those with incomplete information. Finally, we retained 487 patients for the study. The clinicopathological variables associated with PCa in this study cohort are shown in Table 1. IMMPORT (see text footnote 2) is a bioinformatic database for analyses in the field of immunology (Bhattacharya et al., 2014, 2018). We downloaded the list of immunologically relevant genes from it and removed duplicates (shown in Supplementary Table 1). Based on two recently published studies (Li et al., 2020; Zhang et al., 2020), we used the profiling data of mRNAs as well as clinical data in four public datasets (GSE116918, DKF2018, MSKCC2010, and ICGC-PRAD-FR) as external validation sets to validate the effectiveness of the risk predictive model (Gerhauser et al., 2018; Jain et al., 2018; Taylor et al., 2010). We downloaded GSE116918 from the Gene Expression Omnibus (GEO)^4^. We downloaded DKF2018 and MSKCC2010 from cBioPortal for Cancer Genomics^5^, which provides visualization, analysis, and downloading of large-scale cancer genomics datasets (Cerami et al., 2012; Gao et al., 2013). Finally, we downloaded ICGC-PRAD-FR from the International Cancer Genome Consortium (ICGC) database^6^. Information on these four publicly available independent validation datasets is presented in Table 2.

**TABLE 1:** The clinicopathologic variables of patients with PCa included in the study.

Characteristics	Value
Patients (*n*)	487
Age (years), median(IQR)	62.0(56.0−66.0)
**Pathological Gleason score, *n* (%)**	
≤6	43 (8.8)
7 (3 + 4)	143 (29.4)
7 (4 + 3)	101 (20.7)
8	61 (12.5)
9∼10	139 (28.6)
**Prior malignancy, *n* (%)**	
No	459 (94.3)
Yes	28 (5.7)
**Ethnicity, *n* (%)**	
Asian	12 (2.5)
White, American Indian, or Alaska native	406 (83.4)
Black or African American	55 (11.3)
NA	14 (2.8)
**Residual tumor, *n* (%)**	
R0	309 (63.4)
R1	15 (3.1)
R2	144 (29.6)
Rx	5 (1.0)
NA	14 (2.9)
**Clinical M, *n* (%)**	
M0	446 (91.6)
M1a or M1c	2 (0.4)
NA	39 (8.0)
**Pathological T, *n* (%)**	
T1c	2 (0.4)
T2a	13 (2.7)
T2b	10 (2.1)
T2c	161 (33.1)
T3a	157 (32.2)
T3b	132 (27.1)
T4	9 (1.8)
NA	3 (0.6)
**Pathological N, *n* (%)**	
N0	337 (69.2)
N1	79 (16.2)
NA	71 (14.6)
**Diagnostic CT or MRI, *n* (%)**	
No evidence of extraprostatic extension	200 (41.1)
Equivocal	6 (1.2)
Extraprostatic extension localized	22 (4.5)
Extraprostatic extension	9 (1.8)
NA	250 (51.4)
**Outcome, *n* (%)**	
Cancer-specific death or biochemical recurrence	57 (11.7)
Disease free	430 (88.3)

*PCa, prostate cancer; IQR, interquartile range; NA, not analyzed.*

**TABLE 2:** Information of the four publicly available independent validation datasets.

Dataset	Patient Size	Transcriptome Platform	Tissue
GSE1166918	248	ADXPCv1a520642 Affymetrix Human	Formalin-Fixed Paraffin-Embedded
DKFZ2018	82	Illumina HiSeq 2000 (RNAseq)	Fresh frozen
MSKCC2010	140	Affymetrix Human Exon 1.0 ST Array	Fresh frozen
ICGC-PRAD-FR	25	Illumina HiSeq 2000 (RNAseq)	Fresh frozen

### Consensus Clustering in Prostate Cancer Patients

We used the DESeq2 R package to process the RNA-seq data in the form of HTSeq-Counts to identify differentially expressed genes (DEGs) between PCa and normal prostate tissues (Love et al., 2014). We set the screening criteria for differential expression as follows: adjusted *p* < 0.05 and absolute value of the logarithmic fold change (| LFC|) > 1. The adjust method for *p* value was false discovery rate (FDR). We selected genes that were both immunologically relevant genes and DEGs; we called these genes immune DEGs. We then used RNA-seq data in TPM to make an immune DEG matrix for all patients and performed log_2_(*x* + 1) conversion of the data. Then, we employed ConsensusClusterPlus R package to perform consensus clustering analysis of the immune DEG matrix (Monti et al., 2003). The operating parameters were set as follows: maxK = 10, reps = 1,000, pItem = 0.8, pFeature = 1, clusterAlg = “hc,” distance = “pearson,” seed = 1,262,118,388.71279. According to the results of ConsensusClusterPlus, we determined the most consensual cluster subtypes for the patients. We demonstrated the immune DEG expression of 487 patients through a heatmap using the pheatmap R package (Kolde and Kolde, 2015). We then performed survival analysis of the subtypes using the log-rank test with the survival R package (Therneau, 2014). We used disease-free survival (DFS) as the end event and calculated it in the survival analysis. We used the survminer R package to plot the survival curve by the Kaplan–Meier method (Kassambara et al., 2017). Based on the results of survival analysis, we identified high-risk (C3) and low-risk (C1 + C2 + C4) subtypes. We performed principal component analysis on the 487 patients on the basis of the high-risk (C3) and low-risk (C1 + C2 + C4) subtypes using the DESeq2 R package (Love et al., 2014). Then, we used the Mann–Whitney *U* test, χ^2^ test, or Fisher’s exact test to analyze the correlations between clinicopathological variables and subtype status in the 487 patients.

### Determining the Best Consensus Clustering Result

**FIGURE 1:**
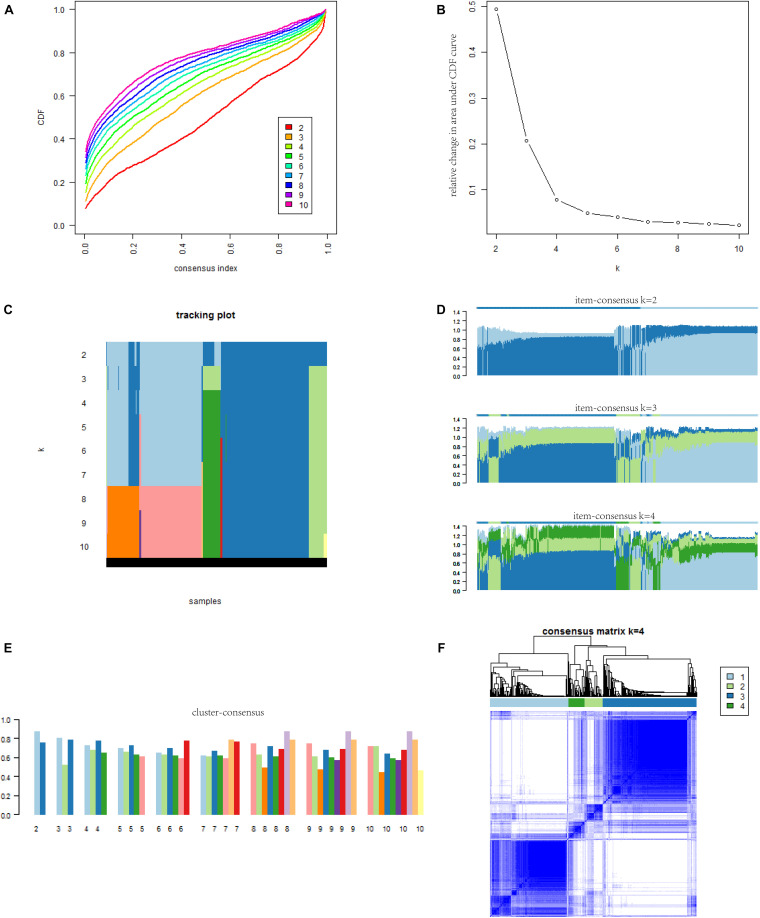
The results of consensus cluster for PCa patients. **(A)** The CDF curve under different values of *k*. The value of *k* represents the number of clusters during the consensus cluster. When the optimal *k* value is reached, the area under the CDF curve will not significantly increase with the increase of *k* value. **(B)** Relative change in area under the CDF curve under different values of *k*. **(C)** The *x*-coordinate of the graph is items, and the *y*-coordinate is the value of *k*. Each color corresponds to different cluster classification colors in the consensus cluster. If items always change the type of cluster (i.e., change the color in a column), it indicates an unstable classification relationship. If a cluster has a large number of samples with unstable classification, it indicates that the cluster is not a stable classification and cannot become a subtyping. **(D)** The figure reflects the item-consensus of each patient for different clusters. The item-consensus reflects the degree of representation of an individual to different clusters. The greater the value, the more representative the individual is of the characteristics of the corresponding cluster. **(E)** The diagram shows the cluster-consensus for each cluster with different *k* values. The cluster-consensus refers to the average value of the consensus matrix of each cluster and represents the degree of consensus of this cluster. The higher the cluster-consensus of the cluster, the higher the stability of this cluster. **(F)** The consensus matrix obtained when *k* = 4. Consistency values range from 0 to 1, 0 means never clustering together (white) and 1 means always clustering together (dark blue). PCa, prostate cancer; CDF, cumulative distribution function.

There were 2,976 DEGs and 1,811 immunologically relevant genes. A total of 263 genes (immune DEGs) overlapped between these two groups. These 263 immune DEGs were used for consensus clustering, the process of which is shown in Figure 1. The ConsensusClusterPlus R package produced a set of results that helped us determine the best grouping scheme. Cumulative distribution function (CDF) reflects the distribution of values in the consensus matrix under different *k* values. When the optimal *k* value is reached, the area under the CDF curve will not significantly increase with increasing *k* value. As shown in Figures 1A,B, when *k* reached 4, the area under the CDF curve did not increase significantly. When *k* took different values, we obtained different clustering patterns, which means that one item might be in different clusters with different *k* values. If items always change their cluster membership (i.e., change the color in a column), this indicates an unstable classification relationship. As shown in Figure 1C, items in this study did not always change their cluster membership. The item-consensus reflects the degree of representation of an individual to different clusters. The greater the value, the more representative the individual is of the characteristics of the corresponding cluster. As shown in Figure 1D, we found that most of the items in the study did not change cluster frequently. The cluster-consensus reflects the average value of the consensus matrix of each cluster, which represents the degree of consensus of this cluster. The higher the cluster-consensus of a cluster, the higher the stability of this cluster. The results of the cluster-consensus when *k* was equal to 5 until 10 are shown in Supplementary Figure 1. As shown in Figure 1E, when *k* = 4, each cluster generally had a high cluster-consensus. Finally, we created a heatmap of the consensus matrix when *k* = 4, as shown in Figure 1F.

### Difference of Somatic Variations Between High-Risk and Low-Risk Groups

We used the data of simple nucleotide variation to explore the difference in somatic variation between the high-risk (C3) and low-risk (C1 + 2 + 4) groups. We analyzed the genes with the top 10 mutation frequencies in the cohort. We used the GenVisR R package to display the mutation details in the waterfall plot (Skidmore et al., 2016). We compared the transcription levels of Speckle-type POZ Protein (*SPOP*) in different subtypes. We used Kruskal–Wallis analysis to compare C1, C2, C3, and C4. We also used Wilcoxon’s test to compare the high-risk (C3) and low-risk (C1 + 2 + 4) groups. Then, we compared the relationship between *SPOP* transcription levels and mutations in the high-risk (C3) and low-risk (C1 + 2 + 4) groups through Wilcoxon’s test.

### Copy Number Alterations, TMPRSS2-ERG Fusion, and Androgen Receptor Scores in Each Subtype

We analyzed copy number alterations (CNA) in all patients among the different subgroups. We analyzed the CNA profile of all genes using the chi-square test. We defined *p* < 0.05 as statistically significant. We then screened for genes with statistically significant differences in CNA among the different subgroups. According to the literature review, we found that prostate cell transformation at an early stage requires Clusterin (*CLU*) silencing (Rizzi and Bettuzzi, 2009). Thus, we next explored the differences in the CNA of *CLU* in the different subgroups, as well as the changes in *CLU* expression. Finally, we explored the correlation between *CLU* expression and its CNA.

*TMPRSS2-ERG* fusion gene is a biological indicator associated with the occurrence of PCa, in cases of which it is the most common type of fusion. Based on integrated analysis of paired-end RNA sequencing and DNA copy number data from TCGA, The Tumor Fusion Gene Data Portal^7^ provides a bona fide fusion list across many tumor types (Yoshihara et al., 2015). With the help of this database, we analyzed the differences of *TMPRSS2-ERG* between the different subtypes. We downloaded the Prostate Adenocarcinoma (TCGA, Cell 2015) dataset in cBiopPortal and obtained the androgen receptor (AR) score of each patient (Cancer Genome Atlas Research Network, 2015). Finally, we explored the differences of AR scores among the different subtypes. In this part of the study, we used the Kruskal–Wallis test to compare C1, C2, C3, and C4 and Wilcoxon test’s to compare C1 + C2 + C4 and C3.

### Infiltration of Immune Cells Into the Tumor Microenvironment in Each Subtype

We analyzed RNA-seq data in TPM format from the 487 patients in CIBERSORTx^8^ (Newman et al., 2019). The parameters were set as follows: signature genes: LM22, batch correction mode: B-mode, and permutations: 100. Then, we demonstrated the infiltration of immune cells into the tumor microenvironment of the patients through a heatmap using the pheatmap R package (Kolde and Kolde, 2015). Next, we represented such infiltration of some patients using a bar plot. We used Wilcoxon’s test to compare the degree of infiltration of 22 kinds of immune cells between the high-risk (C3) and low-risk (C1 + 2 + 4) groups. We considered a *P* value of less than 0.05 as statistically significant.

### Gene Set Enrichment Analysis for Consensus Clusters

We used RNA-seq data in HTSeq-Counts format from the 487 patients for the analysis of the differential expression between C3 (high-risk) and C1 + 2 + 4 (low-risk) using the DESeq2 R package. We used LFC as the sequencing of the gene list in Gene Set Enrichment Analysis (GSEA). We performed GSEA using the clusterProfiler R package (Yu et al., 2012). For the gene list, we chose the Hallmarks gene set downloaded from the Molecular Signatures Database^9^ v7.1 (Subramanian et al., 2005; Liberzon et al., 2011). We set *P* < 0.05 to indicate statistical significance. The adjust method for *p* value was FDR.

### Training the Risk Predictive Model by Machine Learning

We used the term NT-DEGs to describe the genes that were differentially expressed between the normal prostate tissue and prostate tumor tissue. Meanwhile, we used the term cluster-DEGs to describe the genes differentially expressed between the high-risk subtype (C3) and low-risk subtype (C1 + 2 + 4). We obtained the NT-DEGs and cluster-DEGs from RNA-seq data in the form of HTSeq-Counts, calculated using the DESeq2 R package (Love et al., 2014). We set the screening criteria for differential expression as follows: adjusted *p* < 0.05 and |LFC| > 1. Then, we selected genes overlapping between the categories of NT-DEGs and cluster-DEGs for survival analysis by log-rank test and Cox regression. We used DFS as the end event and calculated it in the survival analysis. We chose genes with significant associations with survival for both of these methods (*p* < 0.05). Finally, we used these selected genes as input for training the model. Least absolute shrinkage and selection operator (LASSO) regression is a popular method in machine learning. LASSO makes a feature of variable selection and regularization, while fitting the generalized linear model. Before LASSO, we performed log_2_(*x* + 1) conversion for the TPM of selected genes. We randomly divided the 487 patients into a training set and an internal validation set using the caret R package. Patients in the training set and internal validation set are shown in Supplementary Tables 2 and 3, respectively. We performed LASSO regression using the glmnet R package to train the model (Engebretsen and Bohlin, 2019). In terms of the regression model type, the Cox model was selected. We created heatmaps for the gene signatures in the model using the pheatmap R package (Kolde and Kolde, 2015). Then, we compared the difference in risk scores among the four subtypes by the Kruskal–Wallis test and between the high-risk (C3) group and low-risk (C1 + 2 + 4) group by Wilcoxon’s test.

### Validating the Effectiveness of the Model

Because PCa is a relatively indolent disease, we selected 5 years as the end-point of the follow-up. First, we conducted time-dependent receiver operating characteristic (tdROC) analysis in the training set, internal validation set, and external validation sets to calculate the area under the curve (AUC). We performed tdROC with the help of the survivalROC R package (Heagerty et al., 2013). Then, we completed the survival analysis and created the survival curve in the training set, internal validation set, and external validation sets using the survival and survminer R packages (Therneau, 2014; Kassambara et al., 2017). As DKFZ2018 and GSE116918 recorded the patients’ biochemical recurrence, we conducted survival analysis for these two sets using biochemical recurrence-free survival (BCR). In the other datasets, we used DFS as the end event and calculated it in the survival analysis. Finally, we explored the difference in the risk scores between patients with different survival outcomes in the training set, internal validation set, and external validation sets by Wilcoxon’s test.

Furthermore, we compared the clinical diagnostic value of the predictive model with that of clinical features (PSA and Gleason grade) and a 28-gene hypoxia-related prognostic signature (Yang et al., 2018). PSA and Gleason grade are the main clinical methods to judge the prognosis of patients. We used decision curve analysis (DCA) to evaluate the performance of each indicator (Vickers and Elkin, 2006; Kerr et al., 2016). DCA is a method for evaluating and comparing prediction models that incorporates clinical consequences, requires only the dataset on which the models are tested, and can be applied to models that have either continuous or dichotomous results. The “stdca” function performs DCA for time to event or survival outcomes. We used MASS R package and stdca R code to complete the DCA.

### Statistical Analysis

All of the statistical analyses in this study were completed in R 3.6.3. The statistical methods used in each step and the *p* value thresholds are explained in the corresponding sections.

## Results

### Characteristics of Patients in Different Clusters

**FIGURE 2:**
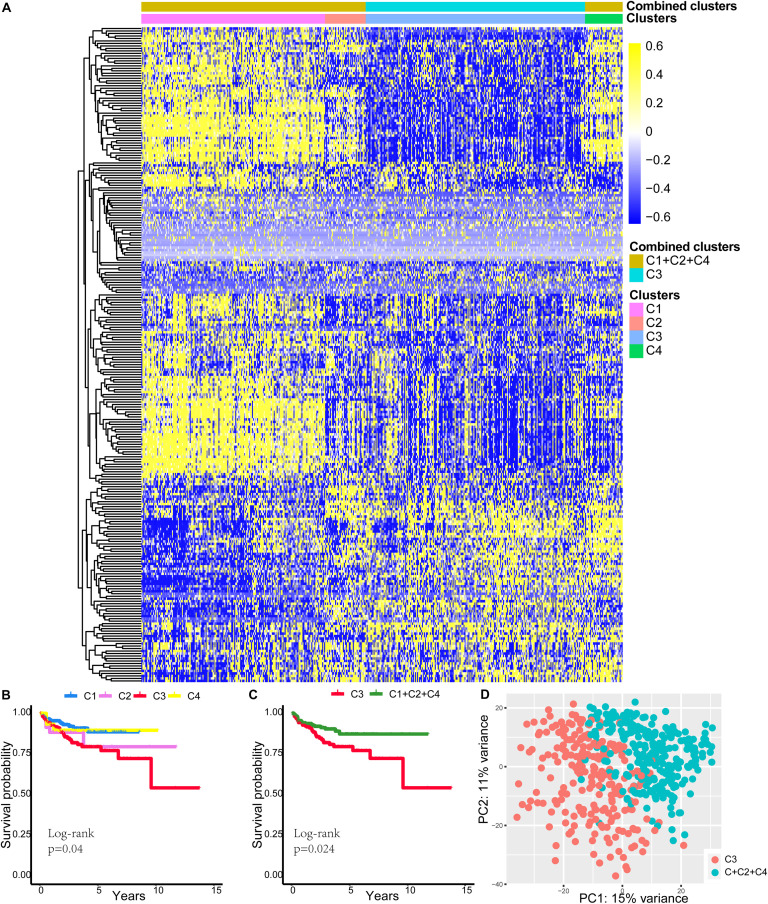
Differences in characteristics of patients in different clusters. **(A)** The heatmap for 263 immune DEG expressions of 487 patients. **(B)** Survival curves for C1, C2, C3, and C4 groups. **(C)** Survival curves for C3 and C1 + 2 + 4 groups. **(D)** Principal component analysis showed that patients in the C3 group were significantly different from patients in the C1 + 2 + 4 group. DEGs, differentially expressed genes.

We eventually divided the 487 patients into four subtypes (C1, C2, C3, and C4). There were 186 patients in the C1 subgroup, 41 in the C2 subgroup, 222 in the C3 subgroup, and 38 in the C4 subgroup. We present the specific subtype grouping of each patient in Supplementary Table 4. The heatmap for the expression of the 263 immune DEGs of the 487 patients is shown in Figure 2A. We found that immune DEGs had different expression patterns among the different subtypes. Survival curves for the C1, C2, C3, and C4 groups are shown in Figure 2B and were found to differ significantly (log-rank test, *p* = 0.04). We found that the survival prognosis of patients in the C3 group was significantly worse than that of the other three groups. Therefore, we combined the C1, C2, and C4 groups and defined them together as a low-risk group, while C3 was defined as a high-risk group. Survival curves for the C3 and C1 + 2 + 4 groups are shown in Figure 2C and were found to differ significantly (log-rank test, *p* = 0.024). Principal component analysis showed that patients in the C3 group were significantly different from those in the C1 + 2 + 4 group (Figure 2D). Furthermore, as shown in Table 3, the PSA level, pathological Gleason score, ethnicity, residual tumor, pathological T, pathological N, and clinical outcome were significantly correlated with the subtype status.

**TABLE 3:** The association between consensus clusters and clinicopathologic variables of prostate cancer.

Clinicopathologic variables	Consensus clusters		*P*
	C1 + C2 + C4 (*n* = 265)	C3 (*n* = 222)	
Age (years), median (IQR)	62.0(57.0−66.0)	61.5(56.0−66.0)	0.228^a^
PSA (ng/ml), median (IQR)	6.65(4.8−10.1)	8.3(5.8−14.1)	< 0.001^a^
**Pathological Gleason score, *n* (%)**			< 0.001^b^
≤6	25 (9.4)	18 (8.1)	
7(3 + 4)	96 (36.2)	47 (21.2)	
7(4 + 3)	59 (22.3)	42 (18.9)	
8	29 (10.9)	32 (14.4)	
9∼10	56 (21.2)	83 (37.4)	
**Prior malignancy, *n* (%)**			0.927^b^
No	250 (94.3)	209 (94.1)	
Yes	15 (5.7)	13 (5.9)	
**Ethnicity, *n* (%)**			0.012^b^
Asian	3 (1.1)	9 (4.1)	
White, American Indian, or Alaska native	232 (87.5)	174 (78.4)	
Black or African American	23 (8.7)	32 (14.4)	
NA	7 (2.7)	7 (3.1)	
**Residual tumor, *n* (%)**			
R0	178 (67.2)	131 (59.0)	0.049^b^
Rx/R1/R2	79 (29.8)	85 (38.3)	
NA	8 (3.0)	6 (2.7)	
**Clinical M, *n* (%)**			0.207^c^
M0	244 (92.1)	202 (91.0)	
M1a or M1c	0 (0)	2 (0.9)	
NA	21 (7.9)	18 (8.1)	
**Pathological T, *n* (%**)			0.048^c^
T1c	2 (0.8)	0 (0.0)	
T2a	8 (3.0)	5 (2.3)	
T2b	5 (1.9)	5 (2.3)	
T2c	101 (38.1)	60 (27.0)	
T3a	82 (30.9)	75 (33.8)	
T3b	63 (23.8)	69 (31.1)	
T4	2 (0.8)	7 (3.2)	
NA	2 (0.7)	1 (0.3)	
**Pathological N, *n* (%)**			0.001^b^
N0	196 (74.0)	141 (63.5)	
N1	30 (11.3)	49 (22.1)	
NA	39 (14.7)	32 (14.4)	
**Diagnostic CT or MRI, *n* (%)**			
No evidence of extraprostatic extension	102 (38.5)	98 (44.1)	0.692^c^
Equivocal	2 (0.8)	4 (1.8)	
Extraprostatic extension localized	9 (3.4)	13 (5.9)	
Extraprostatic extension	4 (1.5)	5 (2.3)	
NA	148 (55.8)	102 (45.9)	
**Outcome, *n* (%)**			
Cancer-specific death or biochemical recurrence	22 (8.3)	35 (15.8)	0.011^b^
Disease free	243 (91.7)	187 (84.2)	

**P* values were calculated by the Mann–Whitney *U* test, chi-square test, or Fisher’s exact test. P < 0.05 was defined as statistically significant. IQR, interquartile range; NA, not analyzed.*

### Difference of Somatic Variations Between High-Risk and Low-Risk Groups

**FIGURE 3:**
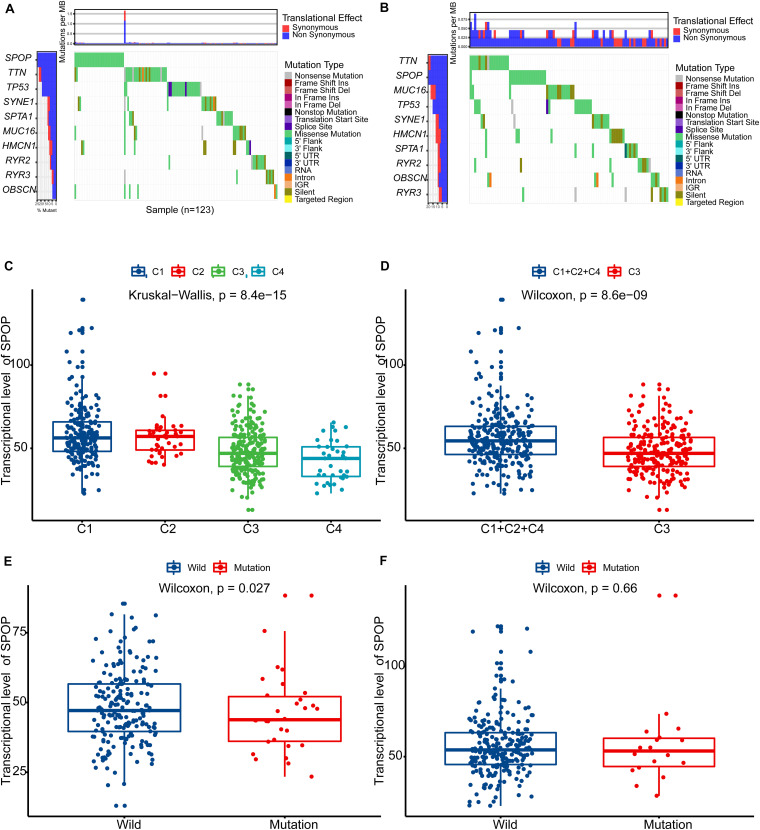
Difference of somatic variations between high-risk (C3) and low-risk (C1 + 2 + 4) groups. **(A)** The map of waterfall for high-risk (C3) group. **(B)** The map of waterfall for low-risk (C1 + 2 + 4) group. **(C)** The differences in the transcription levels of *SPOP* in different subtypes (C1, C2, C3, and C4). **(D)** The differences in the transcription levels of *SPOP* between high-risk (C3) and low-risk (C1 + 2 + 4) groups. **(E)** In the high-risk (C3) group, *SPOP* transcription level of patients with *SPOP* mutation was significantly lower than that of patients with wild-type *SPOP*. **(F)** In the low-risk (C1 + 2 + 4) group, there was no significant difference in *SPOP* transcription between the mutant and the wild-type patients. PCa, prostate cancer; *SPOP*, Speckle-type POZ Protein.

We found that the high-risk (C3) group had higher *SPOP* mutation frequency than the low-risk (C1 + 2 + 4) group (Figures 3A,B). *SPOP* is one of the genes that is most frequently mutated in primary PCa. *SPOP* mutations in PCa are significantly associated with increased PCa cell proliferation and invasion, indicating the loss of function of *SPOP* mutations and the tumor-suppressive role of *SPOP* in PCa (Barbieri et al., 2012; An et al., 2014). Based on this background, we then explored the differences in the transcription levels of *SPOP* in different subtypes. The transcription level of *SPOP* in the C3 group was lower than that in the other groups (Kruskal–Wallis analysis, *p* < 0.01) (Figure 3C), although the *SPOP* transcription level of the C4 group was lower than that of the C3 group. However, owing to the small number of patients in the C4 group, there was a certain selection bias when comparing the differences. As shown in Figure 3D, the *SPOP* transcription level of the C3 (high-risk) group was significantly lower than that of the C1 + 2 + 4 (low-risk) group (Wilcoxon’s test, *p* < 0.01). We found that, in the high-risk (C3) group, the *SPOP* transcription level of patients with *SPOP* mutation was significantly lower than that of patients with wild-type *SPOP* (Wilcoxon’s test, *p* = 0.027) (Figure 3E). However, in the low-risk (C1 + 2 + 4) group, there was no significant difference in *SPOP* transcription between the mutant and wild-type patients (Wilcoxon’s test, *p* = 0.66) (Figure 3F).

### Copy Number Alterations, TMPRSS2-ERG Fusion, and AR Scores in Each Subtype

**FIGURE 4:**
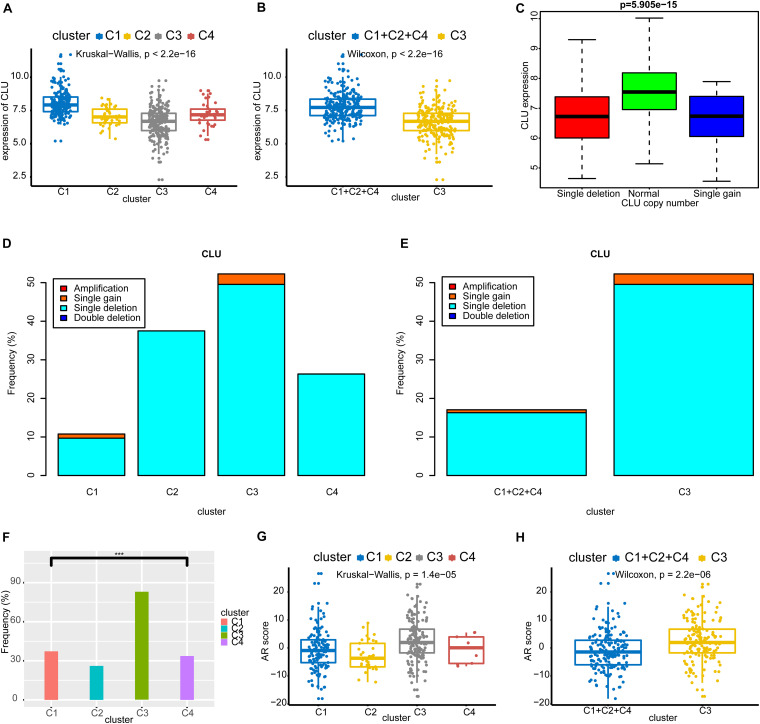
Copy number alterations, *TMPRSS2-ERG* fusion, and AR scores in each subtype. **(A,B)** *CLU* had a lower expression level in C3 subtype. **(C)** The expression level of *CLU* was significantly correlated with its CNA, and the expression level of *CLU* was decreased with single deletion or single gain. **(D,E)** The frequency of CNA in *CLU* in C3 subtype was significantly higher than that in other subtypes. **(F)** C3 had a higher frequency of *TMPRSS2-ERG* fusion. **(G,H)** Patients of the C3 subtype had higher AR scores. CAN, copy number alteration; *CLU*, Clusterin; AR, androgen receptor.

As shown in Figures 4A,B, *CLU* had a lower expression level in the C3 subtype (Kruskal–Wallis test and Wilcoxon’s test, both *p* < 0.001). Consistent with this, *CLU* expression was previously found to be significantly reduced in untreated and hormone-refractory human prostate carcinomas (Rizzi and Bettuzzi, 2009). The expression level of *CLU* was significantly correlated with its CNA, and the expression level of *CLU* was decreased with single deletion or single gain (Figure 4C). We also found that the frequency of CNA in *CLU* in the C3 subtype was significantly higher than that in other subtypes (Figures 4D,E). We also found that C3 had a higher frequency of *TMPRSS2-ERG* fusion (Figure 4F). Finally, we found that patients of the C3 subtype had higher AR scores (Kruskal–Wallis test and Wilcoxon’s test, both *p* < 0.001) (Figures 4G,H).

### Infiltration of Immune Cells Into the Tumor Microenvironment in Each Subtype

**FIGURE 5:**
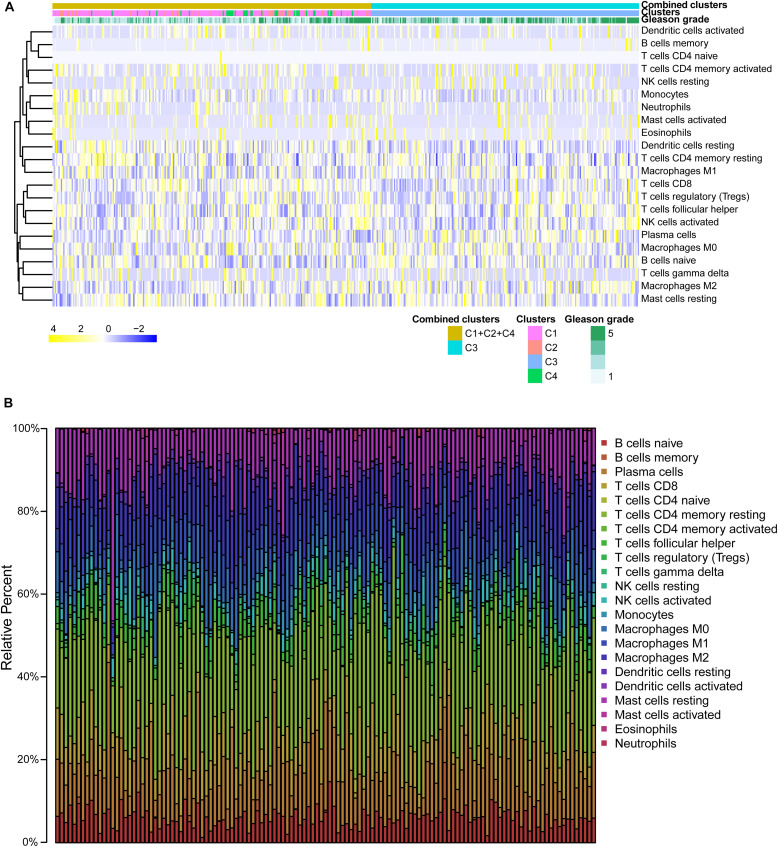
Immune infiltration in each subtype. **(A)** The heatmap about immune infiltration degree of the 487 patients. **(B)** Barplot about immune infiltration of patients.

We presented the infiltration of immune cells into the tumor microenvironment of the 487 patients through a heatmap, as shown in Figure 5A. We found that activated dendritic cells, memory B cells, naïve CD4 T cells, eosinophils, and neutrophils showed little change in infiltration among the groups. Then, we demonstrated the infiltration of immune cells into the tumor microenvironment of the patients in a bar plot. Owing to the large number of patients (*n* = 487), the figure is too large, so for convenience of display, we present only part of this figure in Figure 5B. We found that resting memory CD4 T cells, plasma cells, CD8 T cells, M2 macrophages, and resting mast cells had higher levels of infiltration. We used Wilcoxon’s test to compare the degree of infiltration of 22 kinds of immune cells between the high-risk (C3) and low-risk (C1 + 2 + 4) groups, and the results of which are shown in a violin diagram (Supplementary Figure 2). We found that naïve B cells; plasma cells; and M0, M1, and M2 macrophages infiltrated significantly more into the tumor microenvironment in the high-risk (C3) group. In addition, we found that CD8 T cells, monocytes, resting dendritic cells, activated dendritic cells, and activated mast cells infiltrated significantly more in the low-risk (C1 + 2 + 4) group.

### Gene Set Enrichment Analysis for Consensus Clusters

**FIGURE 6:**
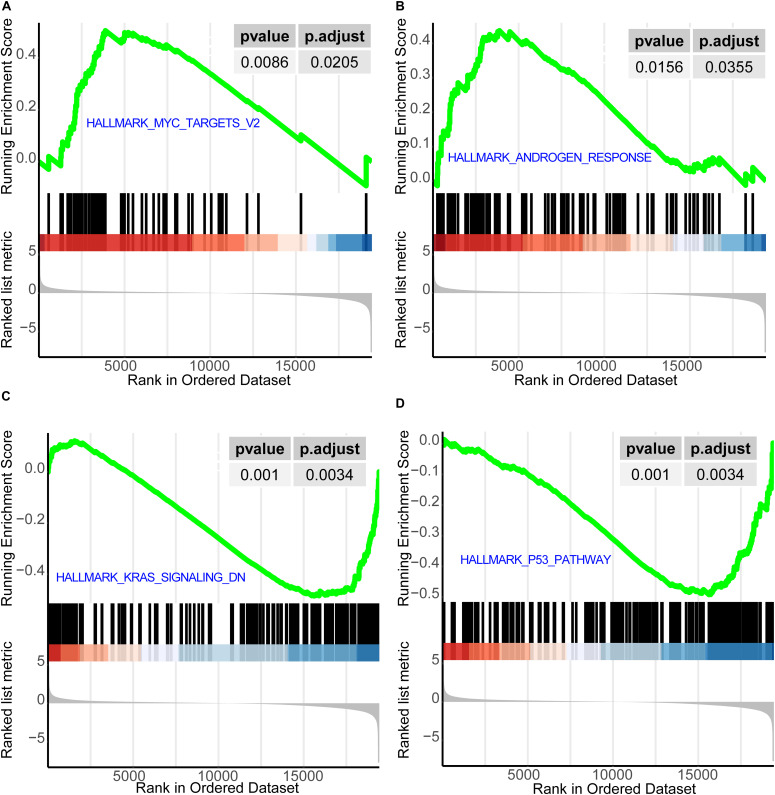
GSEA for consensus clusters. **(A)** HALLMARK_MYC_TARGETS_V2. **(B)** HALLMARK_ANDROGEN_RESPONSE. **(C)** HALLMARK_KRAS_SIGNALING_ DN. **(D)** HALLMARK_P53_PATHWAY. GSEA, Gene Set Enrichment Analysis.

We found that HALLMARK_MYC_TARGETS_V2 and HALLMARK_ANDROGEN_RESPONSE were activated in the high-risk (C3) group (Figures 6A,B), while HALLMARK_ KRAS_SIGNALING_DN and HALLMARK_P53_PATHWAY were relatively suppressed in it (Figures 6C,D). HALLMARK_ MYC_TARGETS_V2 is composed of a subgroup of genes regulated by MYC; HALLMARK_ANDROGEN_RESPONSE is composed of genes defining the response to androgens; HALLMARK_KRAS_SIGNALING_DN is composed of genes downregulated by KRAS activation; and HALLMARK_ P53_PATHWAY is composed of genes involved in p53 pathways and networks. One study overexpressed MYC in the prostate of engineered mice and found that prostatic intraepithelial neoplasia progressed to invasive adenocarcinoma, demonstrating the oncogenic role of MYC in PCa (Ellwood-Yen et al., 2003). The normal development of the prostate requires the presence of androgen; however, androgen can also promote the development of PCa (Heinlein and Chang, 2004). Here, HALLMARK_KRAS_SIGNALING_DN and HALLMARK_P53_PATHWAY were found to be relatively suppressed in the high-risk (C3) group. HALLMARK_ KRAS_SIGNALING_DN is composed of genes that are downregulated when the KRAS signaling pathway is activated. KRAS promotes the development of a variety of tumors including PCa (Chang et al., 2018). In the C3 (high-risk) subtype, these genes are relatively downregulated, which indicates that they are relatively overexpressed in the C1 + 2 + 4 (low-risk) subtype. This indicates that the KRAS signaling pathway is relatively highly activated in the C3 (high-risk) subtype. In support of this, inhibition of the p53 signaling pathway has been reported to facilitate the development of PCa and to contribute to a poor outcome (Takayama et al., 2018).

### The Model Constructed by LASSO Regression

**FIGURE 7:**
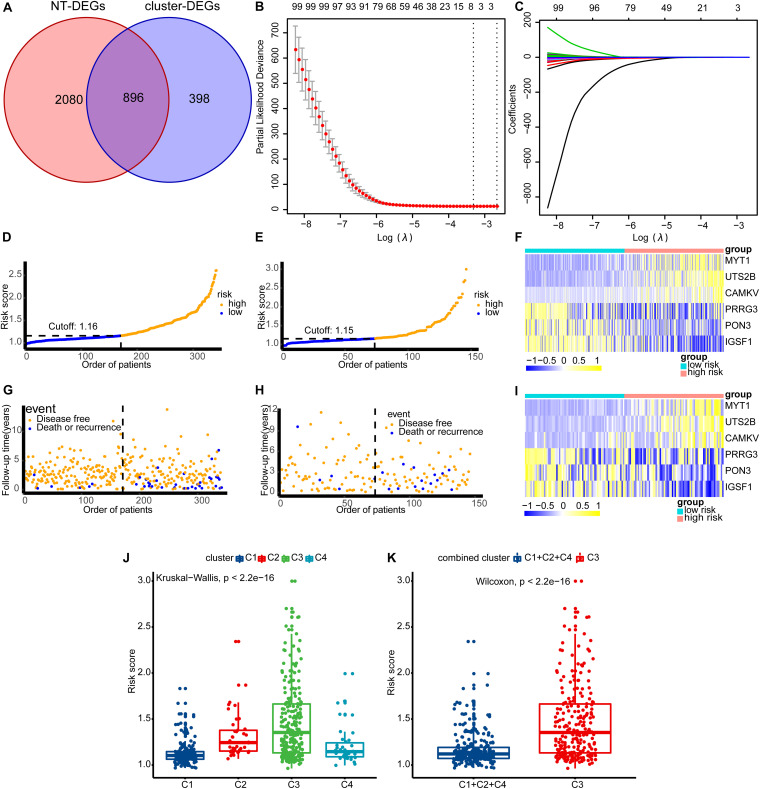
Build the model and see how the model is performing in each dataset. **(A)** The intersection of NT-DEGs and cluster-DEGs served as the input for LASSO regression. **(B)** The λ value corresponding to minimal misclassification error in cross validation. **(C)** Based on the value of λ, the gene signatures and their coefficients of the model were obtained. **(D,G,F)** The results in the training set. **(E,H,I)** The results in the internal validation set. **(D,E)** The distribution of population risk scores and risk classification based on the cut-off value. **(G,H)** The relationship between the survival outcome and risk classification of patients. Low-risk patients are shown on the left side of the dotted line and high-risk patients are shown on the right side. **(F,I)** Heat maps for the gene signatures. **(J)** The difference in risk scores between the four subtypes (C1, C2, C3, and C4). **(K)** The difference in risk scores between the high-risk (C3) group and the low-risk (C1 + 2 + 4) group. NT-DEGs, the differentially expressed genes (DEGs) between the normal prostate tissue and the prostate tumor tissue; cluster-DEGs, the differentially expressed genes (DEGs) between the high-risk subtype (C3) and the low-risk subtype (C1 + 2 + 4); LASSO, least absolute shrinkage and selection operator; λ, punish coefficient.

According to the steps described in the *Training the Risk Predictive Model by Machine Learning* section, we selected a total of 896 genes as input for the survival analysis (Figure 7A). Finally, we selected 104 genes for the LASSO regression. We used cross-validation (10-fold) to find the punish coefficient (λ) to ensure the minimal misclassification error (Figure 7B) (Fan et al., 2019). LASSO regression algorithm would screen the sites in the model and their coefficients based on the λ; we show this process in Figure 7C. We eventually constructed a risk prediction model consisting of six genes (*MYT1*, *UTS2B*, *CAMKV*, *PRRG3*, *PON3*, and *IGSF1*).

$$\begin{array}{c} risks\;core=0.181\times expression\;of\;MYT1+\\ 0.188\times expression\;ofUTS2B+\\ 0.235\times expression\;ofCAMKV-\\ 0.122\times expression\;ofPRRG3-\\ 0.055\times expression\;ofPON3-\\ 0.017\times expression\;ofIGSF1+ \end{array}$$

We then ranked the patients by risk scores in the training set and internal validation set (Figures 7D,E). We then explored the relationship between the survival outcome and risk classification of patients (Figures 7G,H). Low-risk patients are shown on the left side of the dotted line and high-risk patients on the right of it. Heatmaps for the six gene signatures are shown in Figures 7F,I. We found that *MYT1*, *UTS2B*, and *CAMKV* were generally upregulated in high-risk patients, while *PRRG3*, *PON3*, and *IGSF1* were generally downregulated in low-risk patients. Finally, we found that patients in the C3 subtype generally presented a higher risk score (Figures 7J,K) (Kruskal–Wallis test and Wilcoxon’s test, both *p* < 0.01). This is consistent with the results obtained in the process of consensus clustering.

### The Model Demonstrated Good Predictive Performance and More Clinical Benefits

**FIGURE 8:**
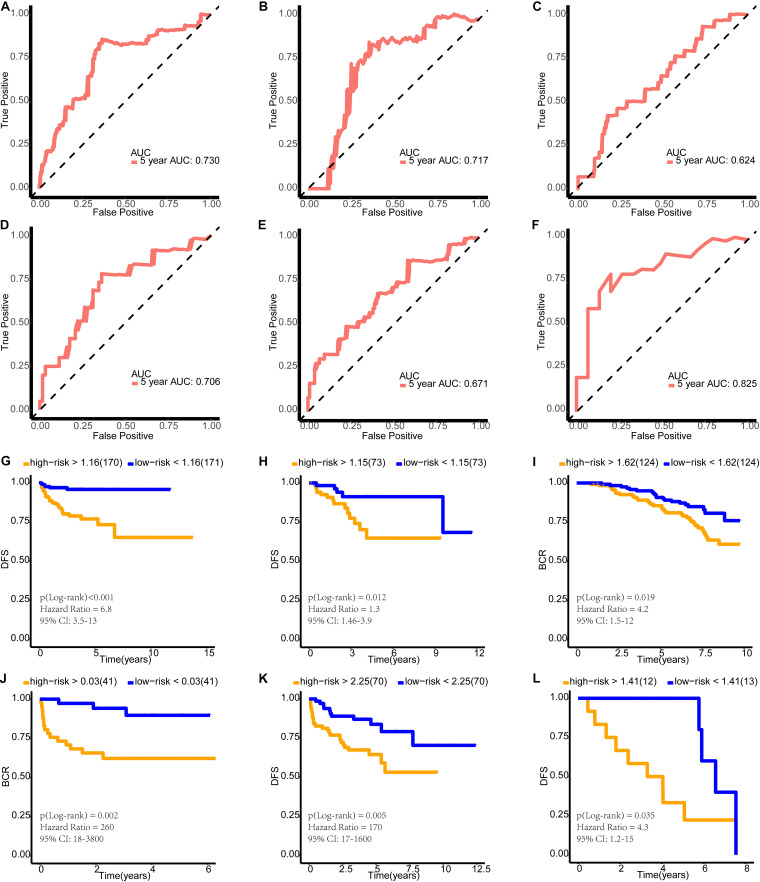
Verification of the effectiveness of the model. **(A–F)** The ROC curve of 5-year follow-up time. **(G–L)** Kaplan–Meier curve for survival analysis. **(A,G)** The results in the training set. **(B,H)** The results in the internal validation set. **(C,I**) The results in GSE116918. **(D,J)** The results in DKFZ2018. **(E,K)** The results in MSKCC2010. **(F,L)** The results in ICGC-PRAD-FR. AUC, area under curve; DFS, disease-free survival; BCR, biochemical recurrence-free survival.

**FIGURE 9:**
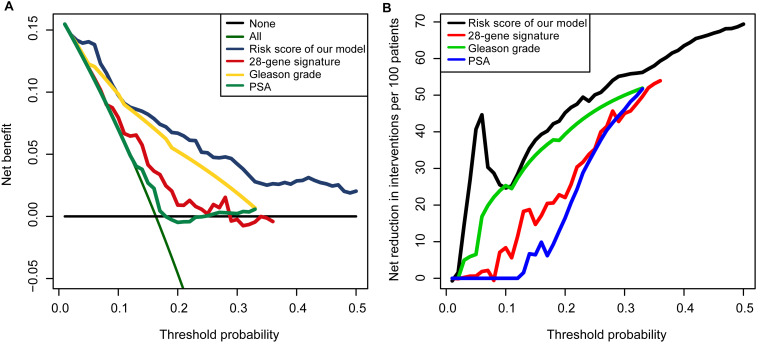
Decision curve analyses suggested that the model had good clinical benefits: **(A)** The model had higher net benefit and wider threshold probability range. The green line is the net benefit of providing all patients with intervention, and the horizontal black line is the net benefit of providing no patients with intervention. **(B)** The net reduction analyses demonstrated in how many patients an intervention could be avoided without missing any poor prognosis.

The AUCs of the tdROC in 5 years were 0.730 in the training set, 0.717 in the internal validation set, 0.624 in GSE116918, 0.706 in DKFZ2018, 0.671 in MSKCC2010, and 0.825 in ICGC-PRAD-FR (Figures 8A–F). Survival analysis and the curves revealed that patients with a risk score greater than the median value had a worse survival prognosis (Figures 8G–L) [training set: *p*(log-rank) < 0.001, HR = 6.8, 95% CI: 3.5–13; internal validation set: *p*(log-rank) = 0.012, HR = 1.3, 95% CI: 1.46–3.9; GSE116918: *p*(log-rank) = 0.019, HR = 4.2, 95% CI: 1.5–12; DKFZ2018: *p*(log-rank) = 0.002, HR = 260, 95% CI: 18–3,800; MSKCC2010: *p*(log-rank) = 0.005, HR = 170, 95% CI: 17–1,600; ICGC-PRAD-FR: *p*(log-rank) = 0.035, HR = 4.3, 95% CI: 1.2–15]. Then, we found that patients with cancer-specific death or biochemical recurrence presented higher risk scores than those with a disease-free status (Supplementary Figure 3) (training set: Wilcoxon, *p* < 0.001; internal validation set: Wilcoxon, *p* = 0.049; GSE116918: Wilcoxon, *p* < 0.0001; DKFZ2018: Wilcoxon, *p* = 0.0016; MSKCC2010: Wilcoxon, *p* = 0.0036; ICGC-PRAD-FR: Wilcoxon, *p* = 0.0011). As shown in Figures 9A,B, the predictive model in this study had a better clinical net benefit and a wider threshold probability range, which suggests that it is more reliable than clinical features (PSA and Gleason grade) and a 28-gene hypoxia-related prognostic signature.

## Discussion

As the most common urinary tumor, the heterogeneity of PCa has been an important topic of research. Exploring novel subtypes of tumors is an effective way of studying their heterogeneity. The immune status of patients can effectively reflect the trends of tumor development and prognosis. Therefore, in this study, we used immune-related genes to conduct consensus clustering among 487 patients and finally identified four PCa subtypes (C1, C2, C3, and C4).

Through survival analysis, we found that the prognosis of patients in the C3 group was significantly worse than that of the other three groups. However, there was no significant difference in prognosis among the three groups C1, C2, and C4. Therefore, we grouped C1, C2, and C4 together and defined them as the low-risk group, while we defined C3 as the high-risk group. Supporting this approach, principal component analysis showed a clear boundary between patients in the high-risk and low-risk groups.

Speckle-type POZ Protein can inhibit the proliferation, migration, and invasion of PCa cells by promoting ATF2 ubiquitination (Ma et al., 2018). PCa-associated *SPOP* mutants are defective at promoting ATF2 degradation in PCa cells and contribute to facilitating PCa cell proliferation, migration, and invasion (Ma et al., 2018). Expressing PCa-associated *SPOP* mutants or knocking out *SPOP* gives PCa cells resistance to cell death caused by stress granule inducers such as docetaxel, sodium arsenite, and H_2_O_2_ (Shi et al., 2019). Strikingly, we found that the high-risk (C3) group had a higher *SPOP* mutation frequency than the low-risk (C1 + 2 + 4) group. Furthermore, the *SPOP* transcription level of the C3 (high-risk) group was significantly lower than that of the C1 + 2 + 4 (low-risk) group (Wilcoxon’s test, *p* < 0.01). In summary, at the somatic variation level, we found that PCa of the high-risk subtype had a higher *SPOP* mutation frequency and lower *SPOP* expression level.

The expression of *CLU* was previously found to be significantly reduced in untreated and hormone-refractory human prostate carcinomas (Rizzi and Bettuzzi, 2009). In this study, *CLU* showed a lower expression level in the C3 subtype. This is consistent with the high-risk characteristics of this subtype. We found that the expression level of *CLU* was significantly correlated with its CNA, and the expression level of *CLU* was decreased with single deletion or single gain. We also found that the frequency of CNA in *CLU* in the C3 subtype was significantly higher than that in other subtypes. This suggested that the low *CLU* expression in the C3 subtype may be related to the CNA status of this gene. We also found that C3 had a higher frequency of *TMPRSS2-ERG* fusion and higher AR score. All of these findings suggested that the C3 subtype is a high-risk phenotype.

We found that naïve B cells; plasma cells; and M0, M1, and M2 macrophages infiltrated significantly more in the high-risk (C3) group. There is increasing evidence that inflammatory cells such as M2 macrophages can promote PCa progression by inhibiting antitumor immune responses (Liang et al., 2016; Cortesi et al., 2018). One study showed that PCa patients with a high number of M2 macrophages in the tumor environment had a significantly increased risk of death from PCa (Erlandsson et al., 2019). The development of resistance to androgen deprivation therapy is also known to be related to the tumor-associated macrophages and neuroendocrine differentiation. Blocking the feedback loop between neuroendocrine differentiation and macrophages was reported to improve the therapeutic effect of enzalutamide on PCa (Wang et al., 2018). T cells can effectively fight against tumors, and this antitumor capacity can be enhanced by immune-regulatory molecular antibodies (checkpoint blockade) (Palucka and Banchereau, 2013). We also found that CD8 T cells, monocytes, resting dendritic cells, activated dendritic cells, and activated mast cells infiltrated significantly more in the low-risk (C1 + 2 + 4) group. CD8^+^ T cells need to be primed and activated toward effector CD8^+^ cytotoxic T lymphocytes, in a process called the tumor immunity cycle, in order to exert durable and efficient antitumor immune responses (Farhood et al., 2019). Dendritic cells are considered a promising weapon for activating the immune system in tumor immunotherapy (Constantino et al., 2017).

After GSEA, we found that HALLMARK_MYC_ TARGETS_V2 and HALLMARK_ANDROGEN_RESPONSE were activated in the high-risk (C3) group. In one study in which MYC was overexpressed in the prostate of engineered mice, it was found that prostatic intraepithelial neoplasia progressed to invasive adenocarcinoma, demonstrating the oncogenic role of MYC in PCa (Ellwood-Yen et al., 2003). The normal development of the prostate requires the presence of androgen; however, androgen can also promote the development of PCa (Heinlein and Chang, 2004). In this study, HALLMARK_KRAS_SIGNALING_DN and HALLMARK_P53_PATHWAY were relatively suppressed in the high-risk (C3) group. HALLMARK_KRAS_SIGNALING_DN is composed of genes that are downregulated when the KRAS signaling pathway is activated. KRAS promotes a variety of tumors including PCa (Chang et al., 2018). In the C3 (high-risk) subtype, these genes are relatively downregulated, which indicates that they are relatively overexpressed in the C1 + 2 + 4 (low-risk) subtype. This indicates that the KRAS signaling pathway is relatively highly activated in the C3 (high-risk) subtype. Inhibition of the p53 signaling pathway facilitates the development of PCa and contributes to a poor outcome (Takayama et al., 2018).

After the establishment of the four subgroups (C1, C2, C3, and C4) and the demonstration of their properties, we constructed a LASSO risk prediction model based on genes differentially expressed between the high-risk and low-risk subgroups. This model consists of six genes: *MYT1*, *UTS2B*, *CAMKV*, *PRRG3*, *PON3*, and *IGSF1*. Based on the expression of these six genes, we could obtain the risk score of individual patients.

Interestingly, the risk coefficients for *MYT1*, *UTS2B*, and *CAMKV* are positive and those for *PRRG3*, *PON3*, and *IGSF1* are negative. *MYT1* is a candidate predictive biomarker of acquired resistance to the Wee1 kinase inhibitor adavosertib (Lewis et al., 2019). Adavosertib has monotherapy activity in a variety of tumors. Cancer cells with intrinsic adavosertib resistance were shown to have higher levels of *MYT1* than sensitive cells. As one of the genes in the model, *UTS2B* has many redundant biological effects with urotensin II. They were shown to be equally potent in stimulating urotensin II receptor, whose mRNA was widely expressed, and notably, their very high levels of transcript were found in the prostate. Christophe et al. found that *UTS2B* may participate more specifically in reproductive functions (Dubessy et al., 2008). In addition, Robyn et al. found that *CAMKV* was not expressed in normal tissues outside of the central nervous system and proposed it as a candidate immunotherapeutic target in *MYCN*-amplified neuroblastoma (Sussman et al., 2020). *PRRG3* is a protein-coding gene, but to the best of our knowledge, no research has explored its role in cancer. Bedİr et al. (2020) found that the *PON3* level decreased significantly in patients with PCa. They also found that *PON3* increased postoperatively in those with PCa. They proposed that surgical excision of malignant tissue in PCa caused a decrease in oxidative stress and that a higher level of *PON3* was associated with lower oxidative stress (Bedİr et al., 2020). Finally, *IGSF1* is a novel oncogene regulating the progression of thyroid cancer (Guan et al., 2019); however, no study describing its role in PCa has been reported. The predictive model established here showed good ability to predict DFS or BCR in TCGA, GSE116918, DKFZ2018, MSKCC2010, and ICGC-PRAD-FR datasets. Furthermore, we compared the clinical diagnostic value of the predictive model with that of clinical features (PSA and Gleason grade) and a 28-gene hypoxia-related prognostic signature. According to the results of DCA, this model had a better clinical net benefit and a wider threshold probability range, which suggests that it is more reliable than current invasive measures. Furthermore, we would like to introduce how to design the model for clinical application: since the model was trained based on RNA-seq data in TPM format, we recommend using the same format of data for evaluation of the prognosis. In order to eliminate the batch effect of detection techniques, we do not recommend other detection techniques to measure gene transcription levels, although we find that the model still performed well on expression profiling by array. In this study, we used the median risk score of the cohort as the threshold for determining high or low risk. In the future, the study cohort should be further expanded to obtain a more objective and stable threshold range before clinical application.

In this study, we first found that PCa patients could be divided into four subtypes (C1, C2, C3, and C4) using immune-related genes. We also found that patients with the C3 subtype had a worse clinical prognosis, so we defined this subtype as the high-risk type. Then, we found that patients of the high-risk (C3) subtype had a higher frequency of *SPOP* mutations. We also revealed that naïve B cells; plasma cells; and M0, M1, and M2 macrophages infiltrated significantly more in the high-risk (C3) group. Moreover, we found that CD8 T cells, monocytes, resting dendritic cells, activated dendritic cells, and activated mast cells infiltrated significantly more in the low-risk (C1 + 2 + 4) group. Finally, we used LASSO regression, a popular machine learning algorithm, to construct a risk prediction model, demonstrating good predictive performance and more clinical benefits, based on the subtype classification. However, the biological mechanisms associated with the subtype classification need to be further explored in future work and the validity of the model needs to be verified in more cohorts.

## Data Availability Statement

The original contributions presented in the study are included in the article/Supplementary Material, further inquiries can be directed to the corresponding author/s.

## Author Contributions

EZ and YS were responsible for the design and conception of the research project, participated in the drafting of the manuscript and the rigorous modification of the manuscript to clearly convey the research contents. EZ, YS, JH, MZ, HZ, LS, and HW contributed to data acquisition, data analysis, and data cleaning. All authors are responsible for the authenticity and reliability of this study and have no objection to the final submitted manuscript.

## Conflict of Interest

The authors declare that the research was conducted in the absence of any commercial or financial relationships that could be construed as a potential conflict of interest.

## Footnotes

1 http://gco.iarc.fr

2 https://www.immport.org/

3 http://asia.ensembl.org/index.html

4 https://www.ncbi.nlm.nih.gov/geo/

5 https://www.cbioportal.org/

6 https://icgc.org/

7 https://www.tumorfusions.org/

8 https://cibersortx.stanford.edu/

9 https://www.gsea-msigdb.org/gsea/msigdb/index.jsp
