# Supplementary material for: Immune-Related Gene-Based Novel Subtypes to Establish a Model Predicting the Risk of Prostate Cancer
Source: Front Genet. 2020 Nov 13;11:595657. doi: 10.3389/fgene.2020.595657 (PMC7691641; doi:10.3389/fgene.2020.595657)
Supplement: Supplementary file 1 [file Data_Sheet_1.docx]

Supplementary Material

# Supplementary Figure 1: For different values of k, the figure reflectes the item-consensus of each patient for different clusters. The item-consensus reflects the degree of representation of an individual to different clusters. The greater the value, the more representative the individual is of the characteristics of the corresponding cluster.


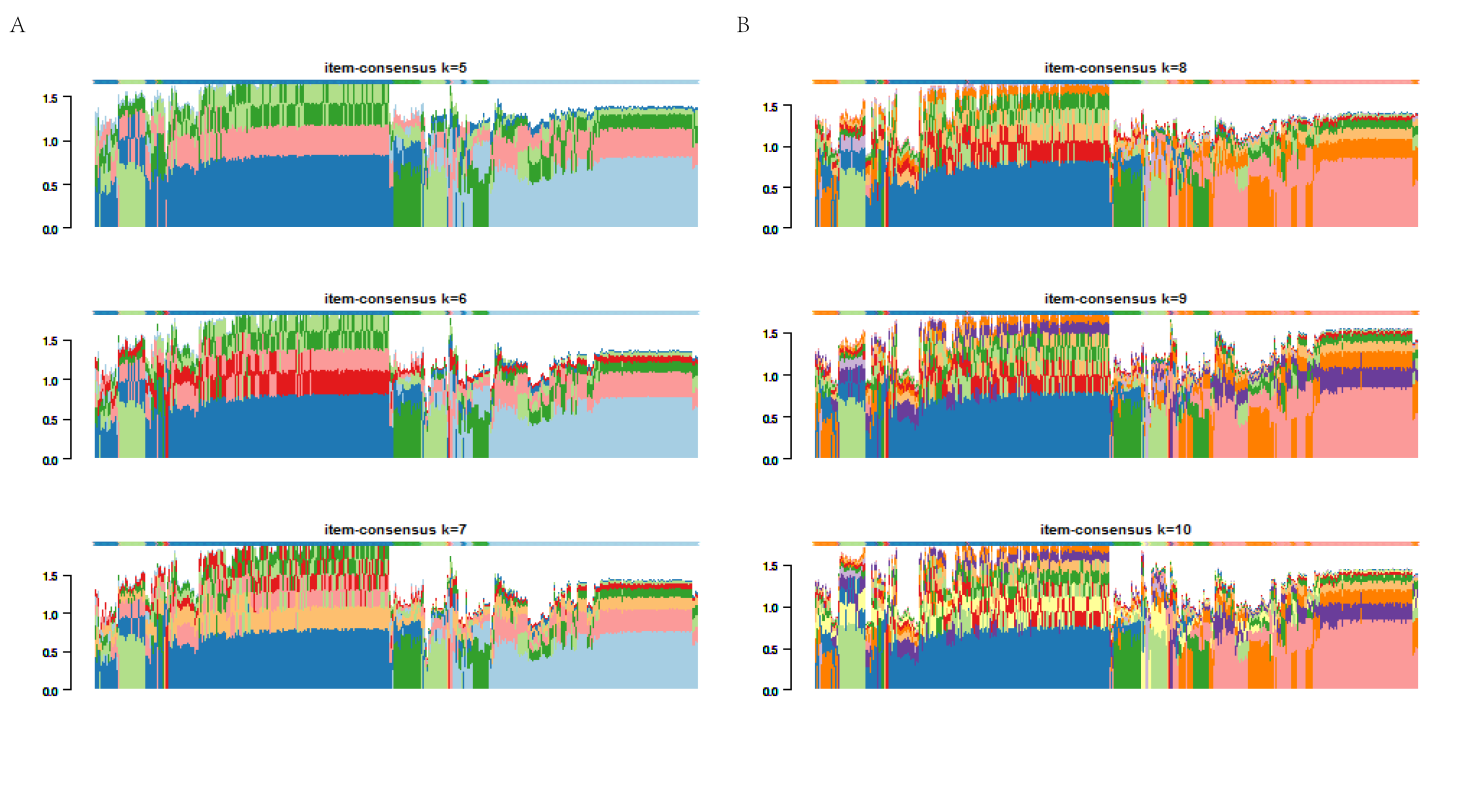


# Supplementary Figure 2: The degree of immune infiltration was different among different subtypes. (A) The violin diagram about infiltration degree of 22 kinds of immune cells between high-risk (C3) and low-risk (C1+2+4) groups.


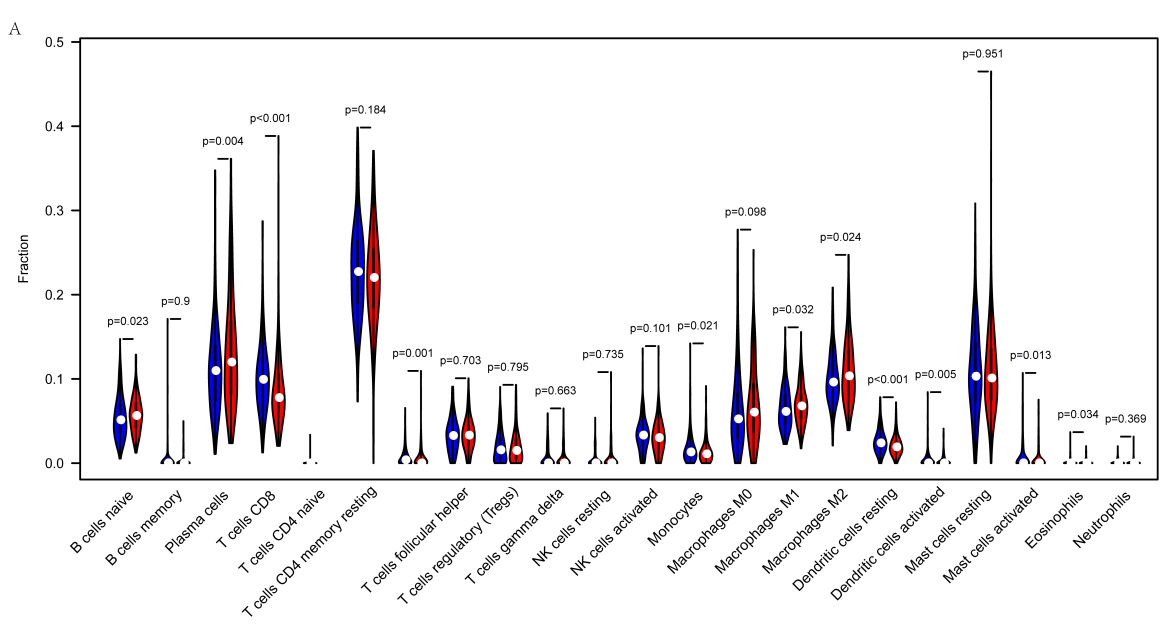


# Supplementary Figure 3: The difference in the risk scores between patients with different survival outcomes. (A) The results in the training set. (B) The results in the internal validation set. (C) The results in GSE116918. (D) The results in DKFZ2018. (E) The results in MSKCC2010. (F) The results in ICGC-PRAD-FR.


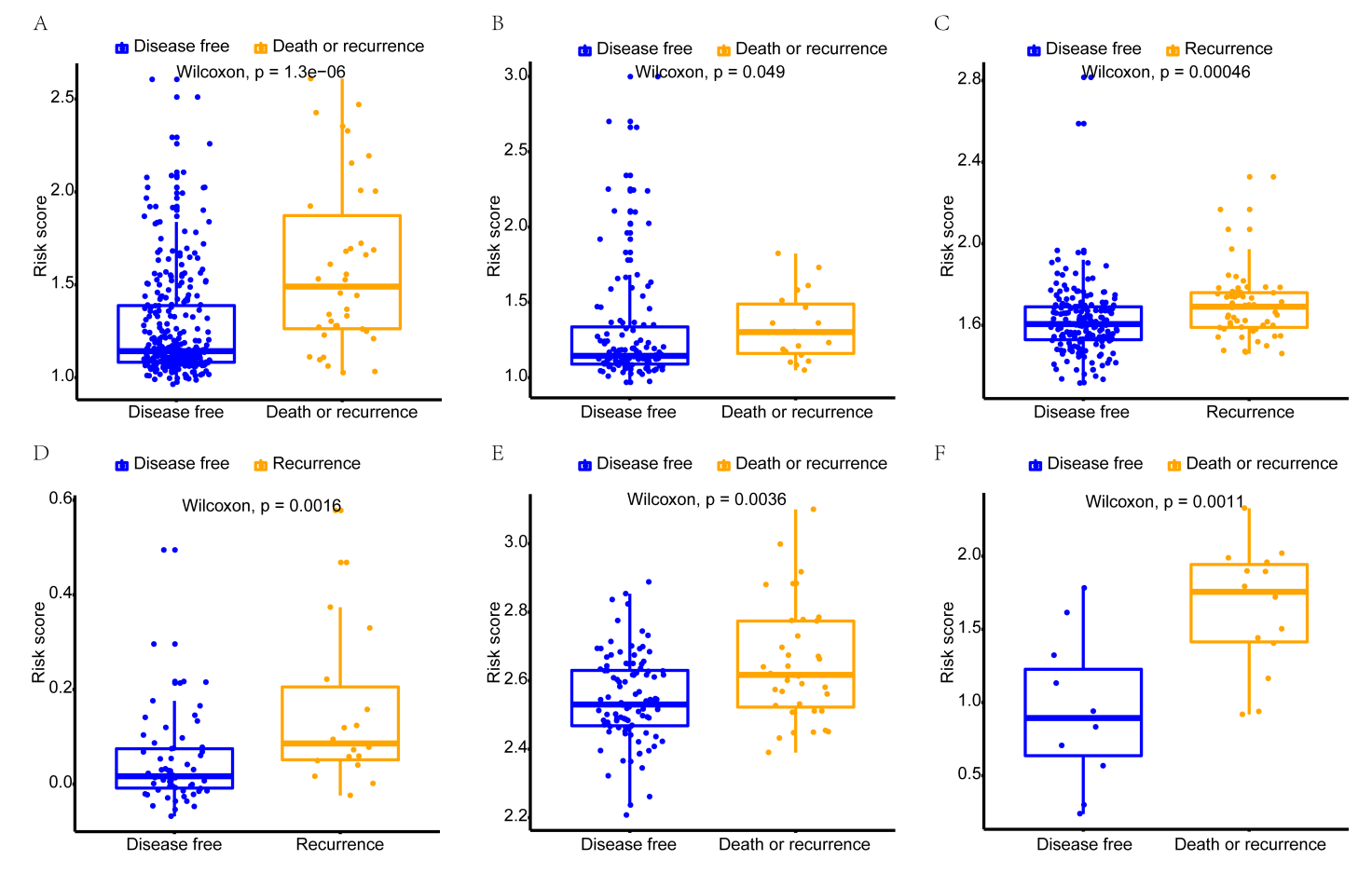


# Supplementary Table1: The immunologically relevant list of genes.

|  | | | | | | | | | |
| --- | --- | --- | --- | --- | --- | --- | --- | --- | --- |
| **Gene Symbol** | | | | | | | | | |
| AZGP1 | LCN1 | PMP2 | PPP4C | IGHD3-3 | IGLV4-69 | FGF4 | TGFA | NR2E3 | TRAJ24 |
| B2M | COLEC10 | APOD | HMOX1 | IGHD3-9 | IGLV5-37 | FGF5 | TGFB2 | NR2F1 | TRAJ25 |
| CALR | BPI | ORM2 | HMGB1 | IGHD4-11 | IGLV5-39 | FGF6 | TGFB3 | NR2F2 | TRAJ26 |
| CANX | S100A9 | ORM1 | RNASE7 | IGHD4-17 | IGLV5-45 | FGF7 | THPO | NR2F6 | TRAJ27 |
| CD1A | S100A8 | TNF | ABCC4 | IGHD4-23 | IGLV5-48 | FGF8 | TNFRSF11B | NR3C1 | TRAJ28 |
| CD1B | DCD | CTSG | HGF | IGHD4-4 | IGLV5-52 | FGF9 | TNFSF12 | NR3C2 | TRAJ29 |
| CD1C | LCN6 | PRTN3 | HDAC1 | IGHD5-12 | IGLV6-57 | FIGF | TNFSF13 | NR4A1 | TRAJ30 |
| CD1D | S100A12 | MAPK1 | IL28RA | IGHD5-18 | IGLV7-43 | FIGNL2 | TNFSF13B | NR4A2 | TRAJ31 |
| CD1E | HTN3 | PML | PLSCR1 | IGHD5-24 | IGLV7-46 | FLT3LG | TNFSF14 | NR4A3 | TRAJ32 |
| CD4 | LCN8 | AEN | BACH2 | IGHD5-5 | IGLV8-61 | FSHB | TNFSF15 | NR5A1 | TRAJ33 |
| CD8A | LOC728358 | CYBB | TANK | IGHD6-13 | IGLV9-49 | GAL | TNFSF18 | NR5A2 | TRAJ34 |
| CD8B | CCR10 | C20orf70 | PIK3CG | IGHD6-19 | C3 | GALP | TNFSF8 | NR6A1 | TRAJ35 |
| CD74 | CELA1 | ISG20 | ARRB1 | IGHD6-25 | C5 | GAST | TNFSF9 | NRP1 | TRAJ36 |
| CREB1 | DEFB106A | BCL3 | RSAD2 | IGHD6-6 | CCL3L2 | GCG | TOR2A | NRP2 | TRAJ37 |
| CTSB | PENK | ISG20L2 | STAB2 | IGHD7-27 | CKLF | GDF1 | TRH | OGFR | TRAJ38 |
| CTSE | BPIL2 | NOX5 | TBK1 | IGHE | CMA1 | GDF10 | TSHB | OPRD1 | TRAJ39 |
| CTSL1 | MMP12 | NOX3 | PDYN | IGHG1 | CX3CL1 | GDF11 | TSLP | OPRK1 | TRAJ40 |
| CTSS | BPIL3 | DUOX2 | PDGFRB | IGHG2 | CXCL17 | GDF2 | TXLNA | OPRL1 | TRAJ41 |
| FCER1G | LEAP2 | TLR3 | PDCD1 | IGHG3 | CYR61 | GDF3 | UCN | OPRM1 | TRAJ42 |
| FCGRT | SFTPD | TFRC | PCSK2 | IGHG4 | EDN1 | GDF5 | UCN2 | OSMR | TRAJ43 |
| PDIA3 | LCN9 | IFIH1 | PCSK1 | IGHJ@ | EDN2 | GDF6 | UCN3 | OXTR | TRAJ44 |
| HFE | BPIL1 | LRP1 | ARG2 | IGHJ1 | EDN3 | GDF7 | UTS2 | PGR | TRAJ45 |
| HLA-A | PTGDS | TRIM5 | AQP9 | IGHJ2 | FGF10 | GDF9 | UTS2D | PGRMC2 | TRAJ46 |
| HLA-B | TMSB4X | IDO1 | FASLG | IGHJ3 | LECT2 | GDNF | VEGFB | PPARA | TRAJ47 |
| HLA-C | PGLYRP1 | GDF15 | APOH | IGHJ4 | PPBPL1 | GH1 | VEGFC | PPARD | TRAJ48 |
| HLA-DMA | ZC3HAV1 | NEDD4 | BIRC5 | IGHJ5 | PROK2 | GH2 | VGF | PRLHR | TRAJ49 |
| HLA-DMB | TMSB15A | ADIPOQ | ANXA6 | IGHJ6 | SAA1 | GHRH | VIP | PRLR | TRAJ50 |
| HLA-DOA | S100B | STAT3 | IL22 | IGHM | SAA2 | GHRL | ACVR1B | PTGER1 | TRAJ52 |
| HLA-DOB | S100A13 | STAT1 | VTN | IGHV@ | SBDS | GIP | ACVR1C | PTGER2 | TRAJ53 |
| HLA-DPA1 | S100A6 | IL28A | VIM | IGHV1-18 | SEMA3A | GKN1 | ACVR2A | PTGER3 | TRAJ54 |
| HLA-DPB1 | DEFB119 | SOCS3 | VCAM1 | IGHV1-2 | SEMA3B | GMFB | ACVR2B | PTGER4 | TRAJ56 |
| HLA-DQA1 | DEFB107A | SEMG1 | PRDX1 | IGHV1-24 | SEMA3C | GMFG | ACVRL1 | PTGFR | TRAJ57 |
| HLA-DQA2 | DEFB105A | TNFSF10 | GFAP | IGHV1-3 | SEMA3D | GNRH1 | ADCYAP1R1 | PTH1R | TRAJ58 |
| HLA-DQB1 | SERPIND1 | CCL20 | GBP2 | IGHV1-45 | SEMA3E | GNRH2 | ADIPOR1 | PTH2R | TRAJ59 |
| HLA-DRA | DEFB129 | SOCS1 | ALB | IGHV1-46 | SEMA3F | GPHA2 | ADIPOR2 | RARA | TRAJ61 |
| HLA-DRB1 | DEFB127 | RNASEL | SLC29A3 | IGHV1-58 | SEMA3G | GPHB5 | ADRB1 | RARB | TRAV1-1 |
| HLA-DRB3 | S100P | IRF1 | OAS1 | IGHV1-69 | SEMA4A | GPI | ADRB2 | RARG | TRAV1-2 |
| HLA-DRB4 | S100A7 | IL15 | AGER | IGHV1-8 | SEMA4B | GREM1 | AGTR1 | RORA | TRAV2 |
| HLA-DRB5 | DEFB104A | APOBEC3F | UNC93B1 | IGHV1-C | SEMA4C | GREM2 | AGTR2 | RORB | TRAV3 |
| HLA-E | DEFB126 | RARRES3 | TNFSF4 | IGHV1-F | SEMA4D | GRP | AMHR2 | RORC | TRAV4 |
| HLA-F | DEFB106B | CHIT1 | NOS1 | IGHV2-26 | SEMA4F | GUCA2A | ANGPT1 | RXFP1 | TRAV5 |
| HLA-G | DEFB104B | CD40 | ACTG1 | IGHV2-5 | SEMA4G | HBEGF | ANGPT4 | RXFP2 | TRAV7 |
| HLA-H | DEFB107B | TLR7 | ACTA1 | IGHV2-70 | SEMA5A | HDGF | ANGPTL1 | RXRA | TRAV8-1 |
| MR1 | PGLYRP3 | PPIA | ACO1 | IGHV3-11 | SEMA5B | HDGFRP3 | ANGPTL2 | RXRB | TRAV8-2 |
| HSPA1A | PGLYRP2 | ZYX | SERPINA3 | IGHV3-13 | SEMA6A | IAPP | ANGPTL3 | RXRG | TRAV8-3 |
| HSPA1B | S100A10 | NLRX1 | IL8RA | IGHV3-15 | SEMA6B | IFNE | ANGPTL4 | S1PR1 | TRAV8-4 |
| HSPA1L | S100A2 | PGC | CCL15 | IGHV3-16 | SEMA6C | IFNK | ANGPTL6 | S1PR2 | TRAV8-6 |
| HSPA2 | DEFB125 | VEGFA | CCL14 | IGHV3-20 | SEMA6D | IFNW1 | APLNR | SCTR | TRAV8-7 |
| HSPA4 | DEFB123 | IKBKE | CCL16 | IGHV3-21 | SEMA7A | IGF1 | AR | SDC1 | TRAV9-1 |
| HSPA5 | DEFB105B | ISG15 | CCL19 | IGHV3-23 | SLIT1 | IGF2 | AVPR1A | SDC2 | TRAV9-2 |
| HSPA6 | DEFB132 | DHX58 | CCL18 | IGHV3-30 | SLIT2 | IL11 | AVPR1B | SDC3 | TRAV10 |
| HSPA8 | C20orf185 | TNFAIP3 | CCL17 | IGHV3-30-3 | TNC | IL12A | AVPR2 | SDC4 | TRAV12-1 |
| HSP90AA1 | LCN12 | TFR2 | CCL26 | IGHV3-30-5 | TYMP | IL16 | BMPR1A | SORT1 | TRAV12-2 |
| HSP90AB1 | PGLYRP4 | FCN2 | CCL22 | IGHV3-33 | C5AR1 | IL17B | BMPR1B | SSTR1 | TRAV12-3 |
| ICAM1 | S100A11 | MUC4 | CCR3 | IGHV3-35 | CCR9 | IL17C | BMPR2 | SSTR2 | TRAV13-1 |
| IFNA1 | S100A5 | F2R | CCL4L2 | IGHV3-38 | CCRL2 | IL17D | BRD8 | SSTR5 | TRAV13-2 |
| IFNA2 | S100A3 | ELN | CCBP2 | IGHV3-43 | CMKLR1 | IL17F | C3AR1 | ST2 | TRAV14DV4 |
| IFNA4 | S100A1 | IL27 | CCR7 | IGHV3-48 | CX3CR1 | IL19 | CALCR | TACR1 | TRAV16 |
| IFNA5 | DEFB128 | MAPT | CCL27 | IGHV3-49 | CXCR3 | IL1F10 | CALCRL | TEK | TRAV17 |
| IFNA6 | DEFB108B | LYZ | CCR8 | IGHV3-53 | CXCR5 | IL1F5 | CNTFR | TGFBR1 | TRAV18 |
| IFNA7 | HTN1 | CCL5 | CCRL1 | IGHV3-64 | CXCR7 | IL1F6 | CRHR1 | TGFBR2 | TRAV19 |
| IFNA8 | LMBR1L | LEP | CCL2 | IGHV3-66 | CYSLTR1 | IL1F7 | CRHR2 | TGFBR3 | TRAV20 |
| IFNA10 | S100A7A | CYLD | CCL21 | IGHV3-7 | CYSLTR2 | IL1F8 | CRIM1 | THRA | TRAV21 |
| IFNA13 | DEFB118 | KLKB1 | CCL7 | IGHV3-72 | DARC | IL1F9 | CRLF1 | THRB | TRAV22 |
| IFNA14 | COLEC12 | CST4 | CCL3 | IGHV3-73 | EDNRA | IL1RN | CRLF2 | TIE1 | TRAV23DV6 |
| IFNA16 | TMSB4Y | CSRP1 | CCL11 | IGHV3-74 | EDNRB | IL20 | CRLF3 | TNFRSF10C | TRAV24 |
| IFNA17 | DEFB131 | MAPK14 | CCR5 | IGHV3-9 | FPR1 | IL21 | CSF1R | TNFRSF10D | TRAV25 |
| IFNA21 | DEFB134 | JUN | CCL23 | IGHV3-D | FPR2 | IL23A | CSF2RA | TNFRSF11A | TRAV26-1 |
| IFNG | DEFB130 | ITGAV | CCL25 | IGHV3-H | GPR17 | IL24 | CSF2RB | TNFRSF12A | TRAV26-2 |
| KIR2DL1 | DEFB124 | IRF5 | CCL3L3 | IGHV4-28 | GPR32 | IL25 | CSF3R | TNFRSF13B | TRAV27 |
| KIR2DL2 | DEFB121 | CCR6 | CCL4L1 | IGHV4-30-1 | GPR33 | IL26 | EGFR | TNFRSF13C | TRAV29DV5 |
| KIR2DL3 | DEFB116 | IL12B | CCL3L1 | IGHV4-30-2 | GPR44 | IL28B | ENG | TNFRSF14 | TRAV30 |
| KIR2DL4 | DEFB115 | TLR8 | CCR1 | IGHV4-30-4 | GPR77 | IL3 | EPOR | TNFRSF17 | TRAV34 |
| KIR2DS1 | DEFB114 | GNLY | CCL24 | IGHV4-31 | IL8RB | IL31 | ESR1 | TNFRSF18 | TRAV35 |
| KIR2DS3 | DEFB113 | CD81 | XCL2 | IGHV4-34 | LTB4R2 | IL32 | ESR2 | TNFRSF19 | TRAV36DV7 |
| KIR2DS4 | DEFB112 | EIF2AK2 | CXCR4 | IGHV4-39 | PLAUR | IL33 | ESRRA | TNFRSF1A | TRAV38-1 |
| KIR2DS5 | DEFB110 | APOM | CXCR6 | IGHV4-4 | PLXNA1 | IL34 | ESRRB | TNFRSF1B | TRAV38-2DV8 |
| KIR3DL1 | TMSB15B | CACYBP | CCR4 | IGHV4-59 | PLXNA2 | IL5 | ESRRG | TNFRSF21 | TRAV39 |
| KIR3DL2 | DEFB133 | NOD1 | FAM19A5 | IGHV4-61 | PLXNA3 | IL6ST | FGFR1 | TNFRSF25 | TRAV40 |
| KLRC1 | S100Z | MAPK8 | FAM19A3 | IGHV4-B | PLXNA4 | IL7 | FGFR2 | TNFRSF4 | TRAV41 |
| KLRC2 | MAVS | MAPK3 | FAM19A4 | IGHV5-51 | PLXNB1 | IL9 | FGFR3 | TNFRSF6B | TRBC1 |
| KLRC3 | TMSL3 | BST2 | FAM19A1 | IGHV5-A | PLXNB2 | INHA | FGFR4 | TNFRSF8 | TRBC2 |
| KLRD1 | S100A14 | BPHL | FAM19A2 | IGHV6-1 | PLXNB3 | INHBA | FGFRL1 | TNFRSF9 | TRBD1 |
| LTA | LCN10 | PLA2G2A | CCL14-CCL15 | IGHV7-4-1 | PLXNC1 | INHBB | FLT1 | TRHR | TRBD2 |
| CIITA | S100A16 | GRN | PTK2B | IGHV7-81 | PLXND1 | INHBC | FLT3 | TSHR | TRBJ1-1 |
| MICA | DEFB137 | NEWENTRY | IL4 | IGK@ | PTAFR | INHBE | FLT4 | TUBB3 | TRBJ1-2 |
| MICB | DEFB136 | PDGFRA | CDH1 | IGKC | ROBO1 | INS | FSHR | VIPR1 | TRBJ1-3 |
| NFYA | DEFB117 | GNAI1 | LTBP1 | IGKDEL | ROBO2 | INS-IGF2 | GALR2 | VIPR2 | TRBJ1-4 |
| NFYB | DEFB111 | WNT5A | IL13 | IGKJ@ | RXFP3 | INSL3 | GALR3 | PTPN11 | TRBJ1-5 |
| NFYC | ZC3HAV1L | FURIN | IL10 | IGKJ1 | XCR1 | INSL4 | GCGR | ICAM2 | TRBJ1-6 |
| LGMN | S100A7L2 | ADAR | IL2 | IGKJ2 | ADM | INSL5 | GHR | ITGAL | TRBJ2-1 |
| PSMB8 | LOC731414 | TYK2 | PPARG | IGKJ3 | ADM2 | INSL6 | GHRHR | ITGB2 | TRBJ2-2 |
| PSMC1 | LOC730963 | NOS2 | FGR | IGKJ4 | AGRP | JAG1 | GHSR | PAK1 | TRBJ2-3 |
| PSMC2 | COLEC2 | TRAF3 | MIF | IGKJ5 | AGT | JAG2 | GIPR | NCR2 | TRBJ2-4 |
| PSMC3 | DEFB4P | TPT1 | CRP | IGKV@ | AMBN | KGFLP1 | GLP1R | TYROBP | TRBJ2-5 |
| PSMC4 | C20orf186 | TPM2 | JAK2 | IGKV1-12 | AMELX | KGFLP2 | GLP2R | LCK | TRBJ2-6 |
| PSMC5 | IFNAR1 | NEO1 | PTK2 | IGKV1-13 | AMH | KITLG | GNRHR | FCGR3A | TRBJ2-7 |
| PSMC6 | AZU1 | AHNAK | PTGDR | IGKV1-16 | ANGPTL5 | KL | GPER | FCGR3B | TRBV2 |
| PSMD1 | LOC729523 | TLR1 | CD86 | IGKV1-17 | ANGPTL7 | LACRT | HNF4A | LOC652578 | TRBV3-1 |
| PSMD2 | LOC100130154 | TK2 | HCK | IGKV1-27 | APLN | LEFTY1 | HNF4G | NCR1 | TRBV4-1 |
| PSMD3 | LOC100134379 | PRDX2 | VDR | IGKV1-33 | AREG | LEFTY2 | HTR3A | NCR3 | TRBV4-2 |
| PSMD4 | LOC100134289 | MX2 | OLR1 | IGKV1-37 | ARMET | LHB | HTR3B | CD247 | TRBV4-3 |
| PSMD5 | LOC100129216 | FGF2 | ADRBK1 | IGKV1-39 | ARMETL1 | LIF | HTR3C | ZAP70 | TRBV5-1 |
| PSMD7 | DEFA1A3 | FGA | TXK | IGKV1-5 | ARTN | LRSAM1 | HTR3D | LCP2 | TRBV5-4 |
| PSMD8 | LOC100131433 | TCF7L2 | RNASE2 | IGKV1-6 | AVP | LTB | HTR3E | LAT | TRBV5-5 |
| PSMD10 | LCN1L1 | F2RL1 | CD79A | IGKV1-8 | BDNF | LTBP2 | IFNGR2 | PLCG1 | TRBV5-6 |
| PSMD11 | S100G | DAK | CD79B | IGKV1-9 | BMP1 | LTBP3 | IGF1R | SH3BP2 | TRBV5-7 |
| PSMD13 | LOC648637 | MSR1 | LYN | IGKV1D-12 | BMP10 | LTBP4 | IGF2R | FYN | TRBV5-8 |
| PSME1 | LOC100130969 | NFKBIZ | SYK | IGKV1D-13 | BMP15 | MDK | IL10RA | SHC2 | TRBV6-1 |
| PSME2 | LOC100133267 | LMBR1 | BTK | IGKV1D-16 | BMP2 | MIA | IL10RB | SHC4 | TRBV6-2 |
| RELB | LOC100133128 | SPINLW1 | BLNK | IGKV1D-17 | BMP3 | MLN | IL11RA | SHC3 | TRBV6-3 |
| RFX5 | LOC100128174 | SRC | VAV3 | IGKV1D-33 | BMP4 | MSTN | IL11RB | SHC1 | TRBV6-4 |
| RFXAP | TCHHL1 | MPO | VAV1 | IGKV1D-37 | BMP5 | NAMPT | IL12RB1 | GRB2 | TRBV6-5 |
| SLC10A2 | TINAGL1 | ELAVL1 | VAV2 | IGKV1D-39 | BMP6 | NDP | IL12RB2 | SOS1 | TRBV6-6 |
| TAP1 | IFNGR1 | ROBO3 | RAC1 | IGKV1D-42 | BMP7 | NENF | IL13RA1 | SOS2 | TRBV6-7 |
| TAP2 | SLC22A17 | SP1 | RAC2 | IGKV1D-43 | BMP8A | NGF | IL13RA2 | ARAF | TRBV6-8 |
| TAPBP | WFIKKN1 | SOD1 | RAC3 | IGKV1D-8 | BMP8B | NMB | IL15RA | BRAF | TRBV6-9 |
| THBS1 | WFDC2 | PDF | PPP3CA | IGKV2-24 | BTC | NODAL | IL15RB | RAF1 | TRBV7-2 |
| SHFM1 | IL6 | DLL4 | PPP3CB | IGKV2-28 | C19orf10 | NOV | IL17RA | HCST | TRBV7-3 |
| KLRC4 | UMODL1 | ECD | PPP3CC | IGKV2-30 | CALCA | NPFF | IL17RB | CD48 | TRBV7-4 |
| AP3B1 | TGFB1 | SLC11A1 | CHP | IGKV2-40 | CALCB | NPPA | IL17RC | CD244 | TRBV7-6 |
| RFXANK | PF4V1 | DMBT1 | PPP3R1 | IGKV2D-24 | CAT | NPPB | IL17RD | PRKCA | TRBV7-7 |
| PSMD6 | MMP9 | TMEM173 | PPP3R2 | IGKV2D-28 | CCK | NPPC | IL17RE | PRKCG | TRBV7-8 |
| PSME3 | KAL1 | SKIV2L | CHP2 | IGKV2D-29 | CD320 | NPY | IL18R1 | SH2D1B | TRBV7-9 |
| PSMD14 | TLR4 | SEMG2 | NFAT5 | IGKV2D-30 | CD70 | NRG1 | IL18RAP | SH2D1A | TRBV9 |
| CLEC4M | SPAG11B | DES | NFATC1 | IGKV2D-40 | CECR1 | NRG2 | IL1R1 | FAS | TRBV10-1 |
| IFI30 | A2M | DCK | NFATC2 | IGKV3-11 | CER1 | NRG3 | IL1R2 | GZMB | TRBV10-2 |
| PROCR | NFKB1 | DAXX | NFATC3 | IGKV3-15 | CGA | NRG4 | IL1RAP | PRF1 | TRBV10-3 |
| ADRM1 | APOBEC3G | TNFRSF10A | NFATC4 | IGKV3-20 | CGB | NRTN | IL1RL1 | CASP3 | TRBV11-1 |
| KIAA0368 | FABP6 | TNFRSF10B | HRAS | IGKV3-7 | CGB1 | NTF3 | IL1RL2 | BID | TRBV11-2 |
| TRPC4AP | NOD2 | EED | KRAS | IGKV3D-11 | CGB2 | NTF4 | IL20RA | CD3D | TRBV11-3 |
| CD209 | MBL2 | CCL4 | NRAS | IGKV3D-15 | CGB5 | NTS | IL20RB | CD3E | TRBV12-3 |
| UBXN1 | SFTPA1B | LIMS1 | FOS | IGKV3D-20 | CGB7 | NUDT6 | IL21R | CD3G | TRBV12-4 |
| ERAP1 | RBP1 | LALBA | CARD11 | IGKV3D-7 | CGB8 | OGN | IL22RA1 | PTPRC | TRBV12-5 |
| TAPBPL | TLR2 | APOBEC3H | BCL10 | IGKV4-1 | CHGA | OSGIN1 | IL22RA2 | ITK | TRBV13 |
| KIR2DL5A | SLC40A1 | TMPRSS6 | MALT1 | IGKV5-2 | CHGB | OSM | IL23R | TEC | TRBV14 |
| ERAP2 | PLAU | SPINK5 | CHUK | IGKV6-21 | CLCF1 | OSTN | IL27RA | NCK1 | TRBV15 |
| ULBP3 | IL1B | MARCO | IKBKB | IGKV6D-21 | CLEC11A | OXT | IL2RA | NCK2 | TRBV16 |
| ULBP2 | PAEP | BECN1 | IKBKG | IGKV6D-41 | CMTM1 | P11 | IL2RB | GRAP2 | TRBV17 |
| ULBP1 | HFE2 | TNFSF11 | NFKBIA | IGL@ | CMTM2 | PDGFA | IL2RG | PAK2 | TRBV18 |
| KIR3DL3 | MUC5AC | KNG1 | NFKBIB | IGLC@ | CMTM3 | PDGFB | IL31RA | PAK3 | TRBV19 |
| RAET1E | OBP2A | CSK | NFKBIE | IGLC1 | CMTM4 | PDGFC | IL3RA | PAK4 | TRBV20-1 |
| RAET1L | PLTP | KLRK1 | CD19 | IGLC2 | CMTM5 | PDGFD | IL4R | PAK6 | TRBV24-1 |
| UBR1 | MX1 | KCNH2 | CR2 | IGLC3 | CMTM6 | PDGFRL | IL5RA | PAK7 | TRBV25-1 |
| RAET1G | DDX58 | JUND | PIK3R5 | IGLC6 | CMTM7 | PGF | IL6R | RHOA | TRBV27 |
| PDIA2 | IL29 | JAK1 | PIK3R1 | IGLC7 | CMTM8 | PMCH | IL9R | CDC42 | TRBV28 |
| HAMP | IRF3 | CLDN4 | PIK3R2 | IGLJ@ | CNTF | PNOC | INSR | CD28 | TRBV29-1 |
| PI3 | SFTPA2 | CCL28 | PIK3R3 | IGLJ1 | CORT | POMC | KDR | ICOS | TRBV30 |
| CAMP | SFTPA2B | RNASE3 | PIK3CA | IGLJ2 | CRH | PPBPL2 | LEPR | MAP3K8 | TRDC |
| DEFB4 | LPA | RN7SL1 | PIK3CB | IGLJ3 | CSF1 | PPY | LGR4 | MAP3K14 | TRDD1 |
| PPBP | LBP | IRF7 | PIK3CD | IGLJ4 | CSF2 | PRL | LGR5 | CTLA4 | TRDD2 |
| REG3G | RBP4 | IREB2 | AKT3 | IGLJ5 | CSF3 | PRLH | LGR6 | CBLC | TRDD3 |
| CXCL14 | SFTPA1 | ILK | AKT1 | IGLJ6 | CSH1 | PROK1 | LHCGR | CBL | TRDJ1 |
| CXCL16 | NOX4 | IL18 | AKT2 | IGLJ7 | CSH2 | PSPN | LIFR | CBLB | TRDJ2 |
| SLPI | LTF | IL17A | GSK3B | IGLV@ | CSHL1 | PTH | LTBR | CDK4 | TRDJ3 |
| IL8 | IFNB1 | LTB4R | INPP5D | IGLV1-36 | CSPG5 | PTH2 | MC1R | RASGRP1 | TRDJ4 |
| CXCL10 | RBP5 | APOBEC3A | CD22 | IGLV1-40 | CTF1 | PTHLH | MC2R | PDK1 | TRDV1 |
| CXCL9 | FABP7 | MASP2 | CD72 | IGLV1-44 | CTGF | PTN | MC3R | PRKCQ | TRDV2 |
| CXCL5 | FABP5 | TRIM27 | PTPN6 | IGLV1-47 | DKK1 | PYY | MC4R | TRAC | TRDV3 |
| CXCL11 | FABP3 | RELA | LILRB3 | IGLV1-50 | EBI3 | QRFP | MCHR1 | TRAJ1 | TRGV9 |
| CXCL6 | FABP2 | IL7R | FCGR2B | IGLV1-51 | EGF | RABEP1 | MCHR2 | TRAJ2 | TRGV8 |
| CXCL1 | FABP4 | IL1A | RASGRP3 | IGLV10-54 | EPGN | RABEP2 | MET | TRAJ3 | TRGV5 |
| CXCL12 | R3HDML | PTX3 | PLCG2 | IGLV11-55 | EPO | REG1A | MLNR | TRAJ4 | TRGV4 |
| CXCL13 | C20orf71 | IFNAR2 | PRKCB | IGLV2-11 | EREG | RETN | MPL | TRAJ5 | TRGV3 |
| CXCL2 | C20orf114 | IFN1@ | IFITM1 | IGLV2-14 | ESM1 | RETNLB | MTNR1A | TRAJ6 | TRGV2 |
| PF4 | OASL | SYTL1 | IGH@ | IGLV2-18 | FAM3B | RLN1 | MTNR1B | TRAJ7 | TRGJP2 |
| XCL1 | CRABP2 | APOBEC3C | IGHA1 | IGLV2-23 | FAM3C | RLN2 | NGFR | TRAJ8 | TRGJP1 |
| CXCL3 | CRABP1 | DDX17 | IGHA2 | IGLV2-33 | FAM3D | RLN3 | NMBR | TRAJ9 | TRGJP |
| DEFB103A | RBP7 | PTGS2 | IGHD | IGLV2-8 | FGF1 | SCG2 | NPR1 | TRAJ10 | TRGJ2 |
| CCL13 | DUOX1 | HTR1A | IGHD@ | IGLV3-1 | FGF11 | SCGB3A1 | NPR3 | TRAJ11 | TRGJ1 |
| CCL1 | OBP2B | 7-Sep | IGHD1-1 | IGLV3-10 | FGF12 | SCT | NR0B1 | TRAJ12 | TRGC2 |
| DEFB1 | RBP2 | CD40LG | IGHD1-14 | IGLV3-12 | FGF13 | SCYE1 | NR0B2 | TRAJ13 | TRGC1 |
| CCL8 | LCN15 | CD14 | IGHD1-20 | IGLV3-16 | FGF14 | SECTM1 | NR1D1 | TRAJ14 | TRAV6 |
| ELANE | CETP | MASP1 | IGHD1-26 | IGLV3-19 | FGF16 | SLURP1 | NR1D2 | TRAJ15 |  |
| DEFB103B | FABP12 | PROC | IGHD1-7 | IGLV3-21 | FGF17 | SPP1 | NR1H2 | TRAJ16 |  |
| DEFA3 | FABP9 | MAP2K2 | IGHD2-15 | IGLV3-22 | FGF18 | SST | NR1H3 | TRAJ17 |  |
| DEFA1 | PLUNC | MAP2K1 | IGHD2-2 | IGLV3-25 | FGF19 | STC1 | NR1H4 | TRAJ18 |  |
| TMSB10 | LCNL1 | HRG | IGHD2-21 | IGLV3-27 | FGF20 | STC2 | NR1I2 | TRAJ19 |  |
| DEFA6 | C8G | NDRG1 | IGHD2-8 | IGLV3-32 | FGF21 | TAC1 | NR1I3 | TRAJ20 |  |
| DEFA5 | SPAG11A | IRF9 | IGHD3-10 | IGLV3-9 | FGF22 | TDGF1 | NR2C1 | TRAJ21 |  |
| DEFA4 | PI15 | TRIM22 | IGHD3-16 | IGLV4-3 | FGF23 | TDGF3 | NR2C2 | TRAJ22 |  |
| LCN2 | NOX1 | LANCL1 | IGHD3-22 | IGLV4-60 | FGF3 | TG | NR2E1 | TRAJ23 |  |

# Supplementary Table 2: Patients in the training set.

| TCGA-KC-A4BN |
| --- |
| TCGA-J4-A83J |
| TCGA-G9-6378 |
| TCGA-HC-7745 |
| TCGA-EJ-A46I |
| TCGA-X4-A8KS |
| TCGA-ZG-A9LU |
| TCGA-KK-A6E3 |
| TCGA-G9-7523 |
| TCGA-HC-7818 |
| TCGA-V1-A9OX |
| TCGA-HC-7738 |
| TCGA-XK-AAJT |
| TCGA-G9-6365 |
| TCGA-CH-5767 |
| TCGA-G9-6336 |
| TCGA-2A-A8VL |
| TCGA-V1-A9O7 |
| TCGA-HC-7737 |
| TCGA-G9-6371 |
| TCGA-HC-A6AQ |
| TCGA-J4-A67T |
| TCGA-KC-A7FE |
| TCGA-YL-A9WJ |
| TCGA-J4-8198 |
| TCGA-HC-A8D1 |
| TCGA-KK-A7B2 |
| TCGA-G9-7509 |
| TCGA-EJ-5502 |
| TCGA-ZG-A9LS |
| TCGA-HC-8260 |
| TCGA-J4-A83M |
| TCGA-EJ-5503 |
| TCGA-EJ-5505 |
| TCGA-HC-7079 |
| TCGA-J4-A67O |
| TCGA-EJ-7786 |
| TCGA-EJ-A65E |
| TCGA-2A-A8VO |
| TCGA-EJ-A46D |
| TCGA-H9-A6BX |
| TCGA-VN-A88O |
| TCGA-EJ-7792 |
| TCGA-HC-8259 |
| TCGA-YL-A8HO |
| TCGA-KK-A7AY |
| TCGA-J4-A67Q |
| TCGA-KC-A4BL |
| TCGA-KC-A7F3 |
| TCGA-EJ-7314 |
| TCGA-FC-A66V |
| TCGA-HC-7742 |
| TCGA-CH-5771 |
| TCGA-G9-6499 |
| TCGA-HC-A8D0 |
| TCGA-HC-7820 |
| TCGA-CH-5743 |
| TCGA-KK-A8IH |
| TCGA-CH-5761 |
| TCGA-J4-A83N |
| TCGA-EJ-7123 |
| TCGA-G9-6370 |
| TCGA-G9-6385 |
| TCGA-HC-A6AS |
| TCGA-EJ-A46H |
| TCGA-HI-7169 |
| TCGA-CH-5748 |
| TCGA-G9-7522 |
| TCGA-QU-A6IM |
| TCGA-VP-A87E |
| TCGA-M7-A720 |
| TCGA-YL-A8SK |
| TCGA-J4-A6M7 |
| TCGA-G9-7519 |
| TCGA-EJ-A7NJ |
| TCGA-J4-8200 |
| TCGA-J4-AATV |
| TCGA-M7-A71Y |
| TCGA-CH-5762 |
| TCGA-G9-6339 |
| TCGA-EJ-5530 |
| TCGA-EJ-A6RC |
| TCGA-CH-5738 |
| TCGA-EJ-5512 |
| TCGA-EJ-5499 |
| TCGA-V1-A8WL |
| TCGA-G9-6343 |
| TCGA-EJ-7321 |
| TCGA-M7-A721 |
| TCGA-KC-A7FD |
| TCGA-KC-A7FA |
| TCGA-YL-A8HJ |
| TCGA-G9-6496 |
| TCGA-EJ-7793 |
| TCGA-V1-A8ML |
| TCGA-XA-A8JR |
| TCGA-EJ-A8FP |
| TCGA-G9-6333 |
| TCGA-EJ-7794 |
| TCGA-VN-A88M |
| TCGA-ZG-A8QX |
| TCGA-EJ-AB20 |
| TCGA-EJ-5508 |
| TCGA-HC-A6AL |
| TCGA-VP-AA1N |
| TCGA-KK-A7B1 |
| TCGA-FC-7961 |
| TCGA-XJ-A83H |
| TCGA-CH-5763 |
| TCGA-HC-8216 |
| TCGA-XJ-A9DI |
| TCGA-EJ-7327 |
| TCGA-EJ-7331 |
| TCGA-V1-A8MU |
| TCGA-2A-AAYO |
| TCGA-VN-A88P |
| TCGA-EJ-8474 |
| TCGA-EJ-A6RA |
| TCGA-EJ-5506 |
| TCGA-HC-8258 |
| TCGA-EJ-7791 |
| TCGA-VN-A88I |
| TCGA-XJ-A9DK |
| TCGA-G9-6384 |
| TCGA-G9-6356 |
| TCGA-G9-6348 |
| TCGA-G9-7510 |
| TCGA-CH-5792 |
| TCGA-G9-6367 |
| TCGA-EJ-5542 |
| TCGA-HC-7736 |
| TCGA-HC-7748 |
| TCGA-HC-A76W |
| TCGA-HC-7740 |
| TCGA-EJ-7785 |
| TCGA-G9-6361 |
| TCGA-J4-A6G1 |
| TCGA-KK-A7AV |
| TCGA-EJ-5522 |
| TCGA-KK-A6E5 |
| TCGA-HC-7230 |
| TCGA-J4-A67R |
| TCGA-CH-5789 |
| TCGA-HC-7819 |
| TCGA-EJ-5531 |
| TCGA-XJ-A83F |
| TCGA-EJ-A8FN |
| TCGA-EJ-A7NH |
| TCGA-ZG-A8QZ |
| TCGA-XQ-A8TB |
| TCGA-J4-AAU2 |
| TCGA-EJ-7315 |
| TCGA-V1-A9OA |
| TCGA-KK-A6DY |
| TCGA-SU-A7E7 |
| TCGA-HC-7231 |
| TCGA-J4-A67L |
| TCGA-KC-A4BR |
| TCGA-EJ-8470 |
| TCGA-HC-A6HX |
| TCGA-CH-5764 |
| TCGA-EJ-5521 |
| TCGA-EJ-7115 |
| TCGA-QU-A6IP |
| TCGA-EJ-5507 |
| TCGA-V1-A9ZK |
| TCGA-YL-A8SO |
| TCGA-Y6-A8TL |
| TCGA-G9-6498 |
| TCGA-QU-A6IL |
| TCGA-EJ-5495 |
| TCGA-CH-5769 |
| TCGA-G9-6342 |
| TCGA-EJ-5509 |
| TCGA-EJ-5510 |
| TCGA-G9-6329 |
| TCGA-KK-A8IJ |
| TCGA-FC-A8O0 |
| TCGA-KK-A6E1 |
| TCGA-G9-6369 |
| TCGA-VN-A88L |
| TCGA-VP-A87C |
| TCGA-J4-A67M |
| TCGA-KK-A8I7 |
| TCGA-EJ-5527 |
| TCGA-WW-A8ZI |
| TCGA-ZG-A9LM |
| TCGA-G9-6354 |
| TCGA-VP-A878 |
| TCGA-CH-5772 |
| TCGA-G9-6364 |
| TCGA-G9-6332 |
| TCGA-J4-A6G3 |
| TCGA-YL-A8SQ |
| TCGA-HC-7817 |
| TCGA-XK-AAK1 |
| TCGA-FC-A6HD |
| TCGA-EJ-A65B |
| TCGA-KK-A7B3 |
| TCGA-YL-A9WY |
| TCGA-G9-6338 |
| TCGA-G9-7525 |
| TCGA-TK-A8OK |
| TCGA-HC-7077 |
| TCGA-HC-8264 |
| TCGA-V1-A9OQ |
| TCGA-CH-5790 |
| TCGA-2A-A8W3 |
| TCGA-EJ-7218 |
| TCGA-VP-A87H |
| TCGA-KK-A8IL |
| TCGA-CH-5739 |
| TCGA-G9-6363 |
| TCGA-HC-8262 |
| TCGA-G9-A9S0 |
| TCGA-EJ-A8FS |
| TCGA-J9-A8CM |
| TCGA-Y6-A9XI |
| TCGA-ZG-A9LN |
| TCGA-EJ-5514 |
| TCGA-EJ-A7NN |
| TCGA-ZG-A9L4 |
| TCGA-EJ-5494 |
| TCGA-HC-7744 |
| TCGA-EJ-7788 |
| TCGA-HC-8261 |
| TCGA-YL-A8SR |
| TCGA-HC-7233 |
| TCGA-M7-A722 |
| TCGA-CH-5737 |
| TCGA-YL-A8HK |
| TCGA-J9-A52C |
| TCGA-XK-AAIV |
| TCGA-EJ-5526 |
| TCGA-ZG-A9L0 |
| TCGA-HC-7081 |
| TCGA-XK-AAJA |
| TCGA-J9-A8CL |
| TCGA-VP-A87J |
| TCGA-XK-AAIR |
| TCGA-G9-6366 |
| TCGA-V1-A8WS |
| TCGA-HC-7747 |
| TCGA-VP-A87B |
| TCGA-J9-A8CK |
| TCGA-J9-A52D |
| TCGA-KK-A6E2 |
| TCGA-HC-7210 |
| TCGA-CH-5741 |
| TCGA-2A-AAYU |
| TCGA-2A-A8VX |
| TCGA-KK-A59Y |
| TCGA-EJ-7312 |
| TCGA-ZG-A9ND |
| TCGA-V1-A8WV |
| TCGA-YL-A8HL |
| TCGA-CH-5740 |
| TCGA-YL-A8S9 |
| TCGA-V1-A9Z9 |
| TCGA-XK-AAJR |
| TCGA-HC-A632 |
| TCGA-HI-7168 |
| TCGA-XK-AAJP |
| TCGA-HC-A9TH |
| TCGA-KK-A59X |
| TCGA-VN-A88R |
| TCGA-KK-A8I4 |
| TCGA-CH-5791 |
| TCGA-MG-AAMC |
| TCGA-V1-A8WW |
| TCGA-VP-A876 |
| TCGA-HC-8257 |
| TCGA-TP-A8TV |
| TCGA-V1-A9ZG |
| TCGA-CH-5744 |
| TCGA-V1-A8MM |
| TCGA-EJ-5504 |
| TCGA-EJ-7325 |
| TCGA-EJ-5501 |
| TCGA-HC-A6AP |
| TCGA-J4-A83I |
| TCGA-HC-8265 |
| TCGA-EJ-7783 |
| TCGA-HC-7749 |
| TCGA-VN-A88N |
| TCGA-HC-7821 |
| TCGA-G9-6362 |
| TCGA-KK-A6E8 |
| TCGA-M7-A725 |
| TCGA-HC-7213 |
| TCGA-XJ-A9DX |
| TCGA-YL-A9WH |
| TCGA-YL-A9WI |
| TCGA-CH-5794 |
| TCGA-HC-A9TE |
| TCGA-EJ-A65F |
| TCGA-HC-7232 |
| TCGA-G9-A9S7 |
| TCGA-J9-A52E |
| TCGA-EJ-5525 |
| TCGA-ZG-A9N3 |
| TCGA-KK-A8I8 |
| TCGA-HI-7171 |
| TCGA-J4-A67N |
| TCGA-YL-A8SL |
| TCGA-KK-A7AU |
| TCGA-EJ-5519 |
| TCGA-M7-A724 |
| TCGA-YL-A8SA |
| TCGA-HC-A48F |
| TCGA-ZG-A8QY |
| TCGA-G9-7521 |
| TCGA-ZG-A9L5 |
| TCGA-YL-A8SC |
| TCGA-CH-5750 |
| TCGA-EJ-7782 |
| TCGA-KK-A59V |
| TCGA-EJ-A7NM |
| TCGA-KK-A8ID |
| TCGA-YL-A8SJ |
| TCGA-ZG-A9L1 |
| TCGA-HC-A6AO |
| TCGA-YL-A8S8 |
| TCGA-EJ-8469 |
| TCGA-J4-AATZ |
| TCGA-KC-A7F5 |
| TCGA-HC-A631 |
| TCGA-YL-A8HM |
| TCGA-HC-7080 |
| TCGA-KK-A8IA |
| TCGA-EJ-A46F |
| TCGA-CH-5788 |
| TCGA-YL-A8SP |
| TCGA-FC-A4JI |
| TCGA-YL-A9WL |
| TCGA-KK-A5A1 |
| TCGA-KK-A7B4 |
| TCGA-V1-A9ZI |
| TCGA-CH-5751 |
| TCGA-ZG-A9KY |
| TCGA-V1-A9O5 |

# Supplementary Table 3: Patients in the validation set.

| TCGA-EJ-A46E |
| --- |
| TCGA-HC-7211 |
| TCGA-XJ-A9DQ |
| TCGA-M7-A723 |
| TCGA-V1-A9OF |
| TCGA-V1-A8WN |
| TCGA-V1-A8MF |
| TCGA-EJ-5532 |
| TCGA-EJ-5517 |
| TCGA-G9-6353 |
| TCGA-J4-A83K |
| TCGA-EJ-5511 |
| TCGA-EJ-7781 |
| TCGA-KK-A6E4 |
| TCGA-V1-A8MG |
| TCGA-HI-7170 |
| TCGA-CH-5745 |
| TCGA-EJ-A65G |
| TCGA-J4-A83L |
| TCGA-FC-7708 |
| TCGA-CH-5768 |
| TCGA-G9-6351 |
| TCGA-J4-A67S |
| TCGA-VP-A872 |
| TCGA-EJ-AB27 |
| TCGA-EJ-A7NK |
| TCGA-EJ-5515 |
| TCGA-J4-A67K |
| TCGA-EJ-7330 |
| TCGA-KK-A8IB |
| TCGA-ZG-A9L6 |
| TCGA-QU-A6IN |
| TCGA-HC-7750 |
| TCGA-XK-AAJU |
| TCGA-V1-A8X3 |
| TCGA-V1-A9OH |
| TCGA-EJ-A7NF |
| TCGA-EJ-A65M |
| TCGA-EJ-A46B |
| TCGA-YL-A8SH |
| TCGA-EJ-7125 |
| TCGA-H9-7775 |
| TCGA-HC-A76X |
| TCGA-KK-A7AZ |
| TCGA-EJ-5498 |
| TCGA-EJ-7318 |
| TCGA-QU-A6IO |
| TCGA-EJ-A7NG |
| TCGA-YL-A8SB |
| TCGA-KK-A8IM |
| TCGA-HC-7752 |
| TCGA-ZG-A8QW |
| TCGA-KK-A8I5 |
| TCGA-EJ-A8FU |
| TCGA-V1-A9OY |
| TCGA-H9-A6BY |
| TCGA-HC-A6HY |
| TCGA-EJ-7328 |
| TCGA-EJ-8468 |
| TCGA-HC-8256 |
| TCGA-KK-A8IG |
| TCGA-KK-A7AQ |
| TCGA-ZG-A9NI |
| TCGA-EJ-7317 |
| TCGA-ZG-A9MC |
| TCGA-CH-5766 |
| TCGA-G9-6494 |
| TCGA-HC-7078 |
| TCGA-CH-5746 |
| TCGA-EJ-A8FO |
| TCGA-HC-7075 |
| TCGA-KK-A7B0 |
| TCGA-VP-A879 |
| TCGA-G9-6347 |
| TCGA-2A-AAYF |
| TCGA-X4-A8KQ |
| TCGA-CH-5765 |
| TCGA-YL-A8SI |
| TCGA-KK-A8IC |
| TCGA-HC-7209 |
| TCGA-ZG-A9L9 |
| TCGA-KK-A6E6 |
| TCGA-EJ-7797 |
| TCGA-ZG-A9L2 |
| TCGA-ZG-A9LY |
| TCGA-CH-5753 |
| TCGA-G9-6379 |
| TCGA-G9-6377 |
| TCGA-KK-A8I6 |
| TCGA-HC-8266 |
| TCGA-KK-A7AW |
| TCGA-4L-AA1F |
| TCGA-EJ-5516 |
| TCGA-EJ-5524 |
| TCGA-YL-A9WX |
| TCGA-V1-A9Z8 |
| TCGA-HC-8213 |
| TCGA-KC-A7F6 |
| TCGA-KK-A6E7 |
| TCGA-2A-A8VV |
| TCGA-J9-A8CN |
| TCGA-VN-A943 |
| TCGA-KC-A4BV |
| TCGA-ZG-A9LZ |
| TCGA-2A-A8W1 |
| TCGA-EJ-7784 |
| TCGA-HC-A6AN |
| TCGA-KK-A59Z |
| TCGA-KK-A8II |
| TCGA-EJ-8472 |
| TCGA-KK-A8I9 |
| TCGA-TP-A8TT |
| TCGA-G9-6373 |
| TCGA-CH-5754 |
| TCGA-XJ-A83G |
| TCGA-VP-A87K |
| TCGA-HC-A4ZV |
| TCGA-XK-AAJ3 |
| TCGA-KK-A6E0 |
| TCGA-HC-A8CY |
| TCGA-KK-A8IF |
| TCGA-EJ-5518 |
| TCGA-VN-A88K |
| TCGA-EJ-A65J |
| TCGA-YL-A9WK |
| TCGA-G9-A9S4 |
| TCGA-VN-A88Q |
| TCGA-HC-7212 |
| TCGA-EJ-A46G |
| TCGA-VP-A87D |
| TCGA-V1-A9OL |
| TCGA-YJ-A8SW |
| TCGA-ZG-A9LB |
| TCGA-2A-A8VT |
| TCGA-XK-AAIW |
| TCGA-KK-A7AP |
| TCGA-EJ-A65D |
| TCGA-J9-A8CP |
| TCGA-VP-A875 |
| TCGA-M7-A71Z |
| TCGA-CH-5752 |
| TCGA-ZG-A9M4 |
| TCGA-J9-A52B |
| TCGA-FC-A5OB |
| TCGA-EJ-7789 |
| TCGA-KK-A8IK |

# Supplementary Table 4: The specific subtype grouping of each patient.

|  | |
| --- | --- |
| TCGA_id | cluster |
| TCGA-2A-A8VL | C1 |
| TCGA-CH-5738 | C1 |
| TCGA-CH-5743 | C1 |
| TCGA-CH-5745 | C1 |
| TCGA-CH-5746 | C1 |
| TCGA-CH-5761 | C1 |
| TCGA-CH-5762 | C1 |
| TCGA-CH-5763 | C1 |
| TCGA-CH-5767 | C1 |
| TCGA-CH-5768 | C1 |
| TCGA-CH-5771 | C1 |
| TCGA-CH-5789 | C1 |
| TCGA-CH-5792 | C1 |
| TCGA-EJ-5495 | C1 |
| TCGA-EJ-5498 | C1 |
| TCGA-EJ-5499 | C1 |
| TCGA-EJ-5501 | C1 |
| TCGA-EJ-5502 | C1 |
| TCGA-EJ-5503 | C1 |
| TCGA-EJ-5506 | C1 |
| TCGA-EJ-5508 | C1 |
| TCGA-EJ-5510 | C1 |
| TCGA-EJ-5512 | C1 |
| TCGA-EJ-5515 | C1 |
| TCGA-EJ-5516 | C1 |
| TCGA-EJ-5522 | C1 |
| TCGA-EJ-5524 | C1 |
| TCGA-EJ-5526 | C1 |
| TCGA-EJ-5527 | C1 |
| TCGA-EJ-5531 | C1 |
| TCGA-EJ-5542 | C1 |
| TCGA-EJ-7115 | C1 |
| TCGA-EJ-7123 | C1 |
| TCGA-EJ-7314 | C1 |
| TCGA-EJ-7315 | C1 |
| TCGA-EJ-7321 | C1 |
| TCGA-EJ-7327 | C1 |
| TCGA-EJ-7328 | C1 |
| TCGA-EJ-7330 | C1 |
| TCGA-EJ-7331 | C1 |
| TCGA-EJ-7781 | C1 |
| TCGA-EJ-7783 | C1 |
| TCGA-EJ-7785 | C1 |
| TCGA-EJ-7786 | C1 |
| TCGA-EJ-7791 | C1 |
| TCGA-EJ-7793 | C1 |
| TCGA-EJ-7794 | C1 |
| TCGA-EJ-7797 | C1 |
| TCGA-EJ-8468 | C1 |
| TCGA-EJ-8470 | C1 |
| TCGA-EJ-A46B | C1 |
| TCGA-EJ-A46D | C1 |
| TCGA-EJ-A46E | C1 |
| TCGA-EJ-A46H | C1 |
| TCGA-EJ-A46I | C1 |
| TCGA-EJ-A65B | C1 |
| TCGA-EJ-A6RA | C1 |
| TCGA-EJ-A6RC | C1 |
| TCGA-EJ-A7NG | C1 |
| TCGA-EJ-A7NH | C1 |
| TCGA-EJ-A7NJ | C1 |
| TCGA-EJ-A7NK | C1 |
| TCGA-EJ-A8FO | C1 |
| TCGA-EJ-A8FP | C1 |
| TCGA-EJ-A8FU | C1 |
| TCGA-EJ-AB20 | C1 |
| TCGA-EJ-AB27 | C1 |
| TCGA-FC-7708 | C1 |
| TCGA-FC-A66V | C1 |
| TCGA-FC-A8O0 | C1 |
| TCGA-G9-6329 | C1 |
| TCGA-G9-6333 | C1 |
| TCGA-G9-6336 | C1 |
| TCGA-G9-6339 | C1 |
| TCGA-G9-6347 | C1 |
| TCGA-G9-6348 | C1 |
| TCGA-G9-6351 | C1 |
| TCGA-G9-6353 | C1 |
| TCGA-G9-6356 | C1 |
| TCGA-G9-6361 | C1 |
| TCGA-G9-6365 | C1 |
| TCGA-G9-6367 | C1 |
| TCGA-G9-6370 | C1 |
| TCGA-G9-6378 | C1 |
| TCGA-G9-6384 | C1 |
| TCGA-G9-6385 | C1 |
| TCGA-G9-6496 | C1 |
| TCGA-G9-6498 | C1 |
| TCGA-G9-6499 | C1 |
| TCGA-G9-7509 | C1 |
| TCGA-G9-7522 | C1 |
| TCGA-H9-A6BX | C1 |
| TCGA-HC-7079 | C1 |
| TCGA-HC-7210 | C1 |
| TCGA-HC-7211 | C1 |
| TCGA-HC-7231 | C1 |
| TCGA-HC-7232 | C1 |
| TCGA-HC-7233 | C1 |
| TCGA-HC-7736 | C1 |
| TCGA-HC-7737 | C1 |
| TCGA-HC-7738 | C1 |
| TCGA-HC-7740 | C1 |
| TCGA-HC-7742 | C1 |
| TCGA-HC-7745 | C1 |
| TCGA-HC-7747 | C1 |
| TCGA-HC-7748 | C1 |
| TCGA-HC-7750 | C1 |
| TCGA-HC-7817 | C1 |
| TCGA-HC-7818 | C1 |
| TCGA-HC-7820 | C1 |
| TCGA-HC-8258 | C1 |
| TCGA-HC-8260 | C1 |
| TCGA-HC-8265 | C1 |
| TCGA-HC-8266 | C1 |
| TCGA-HC-A6HX | C1 |
| TCGA-HC-A76W | C1 |
| TCGA-HC-A8D0 | C1 |
| TCGA-HC-A8D1 | C1 |
| TCGA-HI-7168 | C1 |
| TCGA-HI-7169 | C1 |
| TCGA-HI-7170 | C1 |
| TCGA-J4-A67O | C1 |
| TCGA-J4-A67Q | C1 |
| TCGA-J4-A6G1 | C1 |
| TCGA-J4-A6G3 | C1 |
| TCGA-J4-A83J | C1 |
| TCGA-J4-A83K | C1 |
| TCGA-J4-AATV | C1 |
| TCGA-J9-A52C | C1 |
| TCGA-KC-A4BL | C1 |
| TCGA-KC-A4BR | C1 |
| TCGA-KC-A7F3 | C1 |
| TCGA-KC-A7FE | C1 |
| TCGA-KK-A6E3 | C1 |
| TCGA-KK-A7AV | C1 |
| TCGA-KK-A7B2 | C1 |
| TCGA-KK-A8I7 | C1 |
| TCGA-KK-A8I9 | C1 |
| TCGA-KK-A8IC | C1 |
| TCGA-KK-A8IL | C1 |
| TCGA-M7-A71Y | C1 |
| TCGA-M7-A720 | C1 |
| TCGA-M7-A723 | C1 |
| TCGA-QU-A6IP | C1 |
| TCGA-SU-A7E7 | C1 |
| TCGA-TK-A8OK | C1 |
| TCGA-V1-A8MF | C1 |
| TCGA-V1-A8WL | C1 |
| TCGA-V1-A8WN | C1 |
| TCGA-V1-A9O7 | C1 |
| TCGA-V1-A9OA | C1 |
| TCGA-V1-A9OH | C1 |
| TCGA-V1-A9Z8 | C1 |
| TCGA-V1-A9Z9 | C1 |
| TCGA-V1-A9ZG | C1 |
| TCGA-V1-A9ZK | C1 |
| TCGA-VN-A88I | C1 |
| TCGA-VP-A878 | C1 |
| TCGA-VP-A879 | C1 |
| TCGA-VP-A87C | C1 |
| TCGA-VP-AA1N | C1 |
| TCGA-XA-A8JR | C1 |
| TCGA-XJ-A83H | C1 |
| TCGA-XJ-A9DI | C1 |
| TCGA-XJ-A9DK | C1 |
| TCGA-XJ-A9DQ | C1 |
| TCGA-XK-AAIR | C1 |
| TCGA-XK-AAJR | C1 |
| TCGA-XK-AAJT | C1 |
| TCGA-XK-AAJU | C1 |
| TCGA-XQ-A8TB | C1 |
| TCGA-YL-A8HO | C1 |
| TCGA-YL-A8SB | C1 |
| TCGA-YL-A8SK | C1 |
| TCGA-YL-A9WJ | C1 |
| TCGA-YL-A9WX | C1 |
| TCGA-ZG-A8QW | C1 |
| TCGA-ZG-A9L0 | C1 |
| TCGA-ZG-A9L4 | C1 |
| TCGA-ZG-A9L6 | C1 |
| TCGA-ZG-A9LB | C1 |
| TCGA-ZG-A9LS | C1 |
| TCGA-ZG-A9LU | C1 |
| TCGA-ZG-A9LY | C1 |
| TCGA-ZG-A9MC | C1 |
| TCGA-ZG-A9NI | C1 |
| TCGA-CH-5740 | C2 |
| TCGA-CH-5750 | C2 |
| TCGA-CH-5753 | C2 |
| TCGA-CH-5754 | C2 |
| TCGA-CH-5764 | C2 |
| TCGA-CH-5766 | C2 |
| TCGA-CH-5769 | C2 |
| TCGA-CH-5790 | C2 |
| TCGA-CH-5791 | C2 |
| TCGA-CH-5794 | C2 |
| TCGA-EJ-5507 | C2 |
| TCGA-EJ-5521 | C2 |
| TCGA-EJ-8472 | C2 |
| TCGA-EJ-A7NN | C2 |
| TCGA-G9-6342 | C2 |
| TCGA-G9-6363 | C2 |
| TCGA-G9-6373 | C2 |
| TCGA-G9-6377 | C2 |
| TCGA-G9-7525 | C2 |
| TCGA-HC-7209 | C2 |
| TCGA-HC-7213 | C2 |
| TCGA-HC-7744 | C2 |
| TCGA-HC-8216 | C2 |
| TCGA-J9-A52B | C2 |
| TCGA-J9-A8CK | C2 |
| TCGA-KC-A7F6 | C2 |
| TCGA-KK-A6DY | C2 |
| TCGA-KK-A6E6 | C2 |
| TCGA-KK-A7B1 | C2 |
| TCGA-KK-A8I4 | C2 |
| TCGA-KK-A8I6 | C2 |
| TCGA-KK-A8IH | C2 |
| TCGA-KK-A8IM | C2 |
| TCGA-TP-A8TT | C2 |
| TCGA-VN-A88L | C2 |
| TCGA-VN-A88Q | C2 |
| TCGA-XJ-A83F | C2 |
| TCGA-YL-A8HK | C2 |
| TCGA-ZG-A8QZ | C2 |
| TCGA-ZG-A9LZ | C2 |
| TCGA-ZG-A9N3 | C2 |
| TCGA-2A-A8VO | C3 |
| TCGA-2A-A8VT | C3 |
| TCGA-2A-A8VV | C3 |
| TCGA-2A-A8VX | C3 |
| TCGA-2A-A8W1 | C3 |
| TCGA-2A-A8W3 | C3 |
| TCGA-2A-AAYF | C3 |
| TCGA-2A-AAYO | C3 |
| TCGA-2A-AAYU | C3 |
| TCGA-4L-AA1F | C3 |
| TCGA-CH-5737 | C3 |
| TCGA-CH-5739 | C3 |
| TCGA-CH-5741 | C3 |
| TCGA-CH-5744 | C3 |
| TCGA-CH-5748 | C3 |
| TCGA-CH-5751 | C3 |
| TCGA-CH-5752 | C3 |
| TCGA-CH-5765 | C3 |
| TCGA-CH-5772 | C3 |
| TCGA-CH-5788 | C3 |
| TCGA-EJ-5494 | C3 |
| TCGA-EJ-5504 | C3 |
| TCGA-EJ-5505 | C3 |
| TCGA-EJ-5511 | C3 |
| TCGA-EJ-5514 | C3 |
| TCGA-EJ-5517 | C3 |
| TCGA-EJ-5518 | C3 |
| TCGA-EJ-5519 | C3 |
| TCGA-EJ-5525 | C3 |
| TCGA-EJ-5530 | C3 |
| TCGA-EJ-5532 | C3 |
| TCGA-EJ-7125 | C3 |
| TCGA-EJ-7218 | C3 |
| TCGA-EJ-7312 | C3 |
| TCGA-EJ-7317 | C3 |
| TCGA-EJ-7318 | C3 |
| TCGA-EJ-7782 | C3 |
| TCGA-EJ-7784 | C3 |
| TCGA-EJ-7788 | C3 |
| TCGA-EJ-7789 | C3 |
| TCGA-EJ-7792 | C3 |
| TCGA-EJ-8469 | C3 |
| TCGA-EJ-8474 | C3 |
| TCGA-EJ-A46F | C3 |
| TCGA-EJ-A46G | C3 |
| TCGA-EJ-A65D | C3 |
| TCGA-EJ-A65E | C3 |
| TCGA-EJ-A65F | C3 |
| TCGA-EJ-A65G | C3 |
| TCGA-EJ-A65J | C3 |
| TCGA-EJ-A7NF | C3 |
| TCGA-EJ-A7NM | C3 |
| TCGA-EJ-A8FN | C3 |
| TCGA-FC-7961 | C3 |
| TCGA-FC-A4JI | C3 |
| TCGA-FC-A5OB | C3 |
| TCGA-G9-6332 | C3 |
| TCGA-G9-6338 | C3 |
| TCGA-G9-6343 | C3 |
| TCGA-G9-6354 | C3 |
| TCGA-G9-6362 | C3 |
| TCGA-G9-6364 | C3 |
| TCGA-G9-6366 | C3 |
| TCGA-G9-6369 | C3 |
| TCGA-G9-6371 | C3 |
| TCGA-G9-6494 | C3 |
| TCGA-G9-7519 | C3 |
| TCGA-G9-7521 | C3 |
| TCGA-G9-7523 | C3 |
| TCGA-G9-A9S0 | C3 |
| TCGA-G9-A9S4 | C3 |
| TCGA-G9-A9S7 | C3 |
| TCGA-H9-7775 | C3 |
| TCGA-H9-A6BY | C3 |
| TCGA-HC-7075 | C3 |
| TCGA-HC-7077 | C3 |
| TCGA-HC-7078 | C3 |
| TCGA-HC-7080 | C3 |
| TCGA-HC-7081 | C3 |
| TCGA-HC-7212 | C3 |
| TCGA-HC-7230 | C3 |
| TCGA-HC-7749 | C3 |
| TCGA-HC-7752 | C3 |
| TCGA-HC-7819 | C3 |
| TCGA-HC-7821 | C3 |
| TCGA-HC-8213 | C3 |
| TCGA-HC-8256 | C3 |
| TCGA-HC-8257 | C3 |
| TCGA-HC-8259 | C3 |
| TCGA-HC-8261 | C3 |
| TCGA-HC-8264 | C3 |
| TCGA-HC-A48F | C3 |
| TCGA-HC-A4ZV | C3 |
| TCGA-HC-A631 | C3 |
| TCGA-HC-A632 | C3 |
| TCGA-HC-A76X | C3 |
| TCGA-HC-A8CY | C3 |
| TCGA-HC-A9TE | C3 |
| TCGA-HC-A9TH | C3 |
| TCGA-HI-7171 | C3 |
| TCGA-J4-8198 | C3 |
| TCGA-J4-8200 | C3 |
| TCGA-J4-A67T | C3 |
| TCGA-J4-A6M7 | C3 |
| TCGA-J4-A83I | C3 |
| TCGA-J4-A83L | C3 |
| TCGA-J4-A83M | C3 |
| TCGA-J4-A83N | C3 |
| TCGA-J4-AATZ | C3 |
| TCGA-J4-AAU2 | C3 |
| TCGA-J9-A52D | C3 |
| TCGA-J9-A8CL | C3 |
| TCGA-J9-A8CM | C3 |
| TCGA-J9-A8CN | C3 |
| TCGA-J9-A8CP | C3 |
| TCGA-KC-A4BN | C3 |
| TCGA-KC-A4BV | C3 |
| TCGA-KC-A7F5 | C3 |
| TCGA-KC-A7FA | C3 |
| TCGA-KC-A7FD | C3 |
| TCGA-KK-A59V | C3 |
| TCGA-KK-A59X | C3 |
| TCGA-KK-A59Y | C3 |
| TCGA-KK-A59Z | C3 |
| TCGA-KK-A5A1 | C3 |
| TCGA-KK-A6E0 | C3 |
| TCGA-KK-A6E1 | C3 |
| TCGA-KK-A6E2 | C3 |
| TCGA-KK-A6E4 | C3 |
| TCGA-KK-A6E5 | C3 |
| TCGA-KK-A6E7 | C3 |
| TCGA-KK-A6E8 | C3 |
| TCGA-KK-A7AP | C3 |
| TCGA-KK-A7AQ | C3 |
| TCGA-KK-A7AU | C3 |
| TCGA-KK-A7AZ | C3 |
| TCGA-KK-A7B3 | C3 |
| TCGA-KK-A7B4 | C3 |
| TCGA-KK-A8I5 | C3 |
| TCGA-KK-A8I8 | C3 |
| TCGA-KK-A8IA | C3 |
| TCGA-KK-A8ID | C3 |
| TCGA-KK-A8IF | C3 |
| TCGA-KK-A8IG | C3 |
| TCGA-KK-A8II | C3 |
| TCGA-KK-A8IJ | C3 |
| TCGA-KK-A8IK | C3 |
| TCGA-M7-A71Z | C3 |
| TCGA-M7-A722 | C3 |
| TCGA-M7-A724 | C3 |
| TCGA-M7-A725 | C3 |
| TCGA-MG-AAMC | C3 |
| TCGA-QU-A6IN | C3 |
| TCGA-TP-A8TV | C3 |
| TCGA-V1-A8MG | C3 |
| TCGA-V1-A8ML | C3 |
| TCGA-V1-A8MM | C3 |
| TCGA-V1-A8WS | C3 |
| TCGA-V1-A8WV | C3 |
| TCGA-V1-A8WW | C3 |
| TCGA-V1-A8X3 | C3 |
| TCGA-V1-A9O5 | C3 |
| TCGA-V1-A9OF | C3 |
| TCGA-V1-A9OL | C3 |
| TCGA-V1-A9OQ | C3 |
| TCGA-V1-A9ZI | C3 |
| TCGA-VN-A88K | C3 |
| TCGA-VN-A88M | C3 |
| TCGA-VN-A88N | C3 |
| TCGA-VN-A88O | C3 |
| TCGA-VN-A88P | C3 |
| TCGA-VN-A88R | C3 |
| TCGA-VN-A943 | C3 |
| TCGA-VP-A872 | C3 |
| TCGA-VP-A875 | C3 |
| TCGA-VP-A876 | C3 |
| TCGA-VP-A87B | C3 |
| TCGA-VP-A87D | C3 |
| TCGA-VP-A87E | C3 |
| TCGA-VP-A87J | C3 |
| TCGA-VP-A87K | C3 |
| TCGA-WW-A8ZI | C3 |
| TCGA-XJ-A83G | C3 |
| TCGA-XJ-A9DX | C3 |
| TCGA-XK-AAIV | C3 |
| TCGA-XK-AAIW | C3 |
| TCGA-XK-AAJ3 | C3 |
| TCGA-XK-AAJP | C3 |
| TCGA-XK-AAK1 | C3 |
| TCGA-Y6-A8TL | C3 |
| TCGA-Y6-A9XI | C3 |
| TCGA-YJ-A8SW | C3 |
| TCGA-YL-A8HJ | C3 |
| TCGA-YL-A8HL | C3 |
| TCGA-YL-A8HM | C3 |
| TCGA-YL-A8S8 | C3 |
| TCGA-YL-A8S9 | C3 |
| TCGA-YL-A8SA | C3 |
| TCGA-YL-A8SC | C3 |
| TCGA-YL-A8SH | C3 |
| TCGA-YL-A8SI | C3 |
| TCGA-YL-A8SJ | C3 |
| TCGA-YL-A8SL | C3 |
| TCGA-YL-A8SP | C3 |
| TCGA-YL-A8SQ | C3 |
| TCGA-YL-A8SR | C3 |
| TCGA-YL-A9WH | C3 |
| TCGA-YL-A9WI | C3 |
| TCGA-YL-A9WK | C3 |
| TCGA-YL-A9WL | C3 |
| TCGA-YL-A9WY | C3 |
| TCGA-ZG-A8QX | C3 |
| TCGA-ZG-A8QY | C3 |
| TCGA-ZG-A9KY | C3 |
| TCGA-ZG-A9L1 | C3 |
| TCGA-ZG-A9L2 | C3 |
| TCGA-ZG-A9L5 | C3 |
| TCGA-ZG-A9L9 | C3 |
| TCGA-ZG-A9LM | C3 |
| TCGA-ZG-A9LN | C3 |
| TCGA-ZG-A9M4 | C3 |
| TCGA-ZG-A9ND | C3 |
| TCGA-EJ-5509 | C4 |
| TCGA-EJ-7325 | C4 |
| TCGA-EJ-A65M | C4 |
| TCGA-EJ-A8FS | C4 |
| TCGA-FC-A6HD | C4 |
| TCGA-G9-6379 | C4 |
| TCGA-G9-7510 | C4 |
| TCGA-HC-8262 | C4 |
| TCGA-HC-A6AL | C4 |
| TCGA-HC-A6AN | C4 |
| TCGA-HC-A6AO | C4 |
| TCGA-HC-A6AP | C4 |
| TCGA-HC-A6AQ | C4 |
| TCGA-HC-A6AS | C4 |
| TCGA-HC-A6HY | C4 |
| TCGA-J4-A67K | C4 |
| TCGA-J4-A67L | C4 |
| TCGA-J4-A67M | C4 |
| TCGA-J4-A67N | C4 |
| TCGA-J4-A67R | C4 |
| TCGA-J4-A67S | C4 |
| TCGA-J9-A52E | C4 |
| TCGA-KK-A7AW | C4 |
| TCGA-KK-A7AY | C4 |
| TCGA-KK-A7B0 | C4 |
| TCGA-KK-A8IB | C4 |
| TCGA-M7-A721 | C4 |
| TCGA-QU-A6IL | C4 |
| TCGA-QU-A6IM | C4 |
| TCGA-QU-A6IO | C4 |
| TCGA-V1-A8MU | C4 |
| TCGA-V1-A9OX | C4 |
| TCGA-V1-A9OY | C4 |
| TCGA-VP-A87H | C4 |
| TCGA-X4-A8KQ | C4 |
| TCGA-X4-A8KS | C4 |
| TCGA-XK-AAJA | C4 |
| TCGA-YL-A8SO | C4 |
